# Comprehensive evaluation of differential gene expression analysis methods for RNA-seq data

**DOI:** 10.1186/gb-2013-14-9-r95

**Published:** 2013-09-10

**Authors:** Franck Rapaport, Raya Khanin, Yupu Liang, Mono Pirun, Azra Krek, Paul Zumbo, Christopher E Mason, Nicholas D Socci, Doron Betel

**Affiliations:** 1 Bioinformatics Core, Memorial Sloan-Kettering Cancer Center, New York, NY, 10065, USA; 2 Department of Physiology and Biophysics, Weill Cornell Medical College, New York, NY, 10021, USA; 3 Institute for Computational Biomedicine, Weill Cornell Medical College, New York, NY, 10021, USA; 4 Division of Hematology/Oncology, Department of Medicine, Weill Cornell Medical College, New York, NY, 10021, USA

## Abstract

A large number of computational methods have been developed for analyzing differential gene expression in RNA-seq data. We describe a comprehensive evaluation of common methods using the SEQC benchmark dataset and ENCODE data. We consider a number of key features, including normalization, accuracy of differential expression detection and differential expression analysis when one condition has no detectable expression. We find significant differences among the methods, but note that array-based methods adapted to RNA-seq data perform comparably to methods designed for RNA-seq. Our results demonstrate that increasing the number of replicate samples significantly improves detection power over increased sequencing depth.

## Background

High-throughput sequencing technology is rapidly becoming the standard method for measuring RNA expression levels (aka RNA-seq) [1]. The advent of rapid sequencing technologies along with reduced costs has enabled detailed profiling of gene expression levels, impacting almost every field in life sciences and is now being adopted for clinical use [2]. RNA-seq technology enables the detailed identification of gene isoforms, translocation events, nucleotide variations and post-transcriptional base modifications [3]. One of the main goals of these experiments is to identify the differentially expressed genes in two or more conditions. Such genes are selected based on a combination of expression change threshold and score cutoff, which are usually based on *P *values generated by statistical modeling.

The expression level of each RNA unit is measured by the number of sequenced fragments that map to the transcript, which is expected to correlate directly with its abundance level. This measure is fundamentally different from gene probe-based methods, such as microarrays. In RNA-seq the expression signal of a transcript is limited by the sequencing depth and is dependent on the expression levels of other transcripts, whereas in array-based methods probe intensities are independent of each other. This, as well as other technical differences, has motivated the development of a growing number of statistical algorithms that implement a variety of approaches for normalization and differential expression (DE) detection. Typical approaches use Poisson or negative binomial distributions to model the gene count data and a variety of normalization procedures (see [4] for a review).

In this comparison study, we evaluated a few of the most commonly used and freely available differential expression software packages: Cuffdiff [5], edgeR [6], DESeq [7], PoissonSeq [8], baySeq [9], and limma [10] adapted for RNA-seq use. We used two benchmark datasets: the first is the Sequencing Quality Control (SEQC) dataset, which includes replicated samples of the human whole body reference RNA and human brain reference RNA along with RNA spike-in controls. These samples are part of the MAQC study for benchmarking microarray technology [11,12] as well as the SEQC effort to characterize RNA-seq technology and include close to 1,000 genes that were validated by TaqMan qPCR. The second dataset is RNA-seq data from biological replicates of three cell lines that were characterized as part of the ENCODE project [13]. Our analysis focused on a number of measures that are most relevant for detection of differential gene expression from RNA-seq data: i) normalization of count data; ii) sensitivity and specificity of DE detection; iii) performance on the subset of genes that are expressed in one condition but have no detectable expression in the other condition and, finally, iv) the effects of reduced sequencing depth and number of replicates on the detection of differential expression. Importantly, this evaluation does not address the related and important problem of detecting differential isoform expression and identification of novel transcripts. Rather, the evaluation is restricted to the specific case of detecting DE based on unified gene models.

Our results demonstrate substantial differences among the methods both in terms of specificity and sensitivity for the detection of differentially expressed genes. In most benchmarks Cuffdiff performed less favorably with a higher number of false positives without any increase in sensitivity. Our results conclusively demonstrate that the addition of replicate samples provides substantially greater detection power of DE than increased sequence depth. Hence, including more replicate samples in RNA-seq experiments is always to be preferred over increasing the number of sequenced reads.

### Theoretical background

A convenient starting point for comparing different RNA-seq analysis methods is a simple count matrix **N **of *n *× *m *where *N_ij _*is the number of reads assigned to gene *i *in sequencing experiment *j *(that is, read counts). Such matrices can be produced from alignment data using tools such as HTSeq [15], Picard [16], BEDTools [17], featureCounts [18] or Cufflinks [19]. The study presented here does not address the important subtleties when calculating gene counts, in particular which gene model to use, how to count reads overlapping intronic regions and the use of ambiguously mapped reads. Rather, the focus is on the comparison between methods given a fixed expression count matrix. For Cuffdiff, which uses a different quantitation method that is not compatible with the others, we used its joint method Cufflinks and for all other methods we used HTSeq. It is important to recognize that the number of reads which overlap a gene *i *is not a direct measure of the gene's expression. Rather the count measure $N_{ij}\propto l_{i}\mu_{ij}$ where *μ_ij _*and *l_i _*are the expected expression and gene length, respectively. Hence there is a clear length bias when measuring gene expression by RNA-seq [20]. One effect of this bias is to reduce the ability to detect differential expression among shorter genes simply from the lack of coverage since the power of statistical tests involving count data decreases with a lower number of counts [21,22].

Differential gene expression analysis of RNA-seq data generally consists of three components: normalization of counts, parameter estimation of the statistical model and tests for differential expression. In this section we provide a brief background into the approaches implemented by the various algorithms that perform these three steps. We limit our discussion to the most common case of measuring differential expression between two cellular conditions or phenotypes although some of the packages can test for multi-class differences or multi-factored experiments where multiple biological conditions and different sequencing protocols are included.

#### Normalization

The first difficulty to address when working with sequencing data is the large differences in the number of reads produced between different sequencing runs as well as technical biases introduced by library preparation protocols, sequencing platforms and nucleotide compositions [23]. Normalization procedures attempt to account for such differences to facilitate accurate comparisons between sample groups. An intuitive normalization is to divide the gene count simply by the total number of reads in each library, or mapped reads, as first introduced by Mortazavi *et al*. [1], a normalization procedure named reads per kilobase per million reads (RPKM). A deficiency of this approach is that the proportional representation of each gene is dependent on the expression levels of all other genes. Often a small fraction of genes account for large proportions of the sequenced reads and small expression changes in these highly expressed genes will skew the counts of lowly expressed genes under this scheme. This can result in erroneous differential expression [24,25]. A variation of RPKM, termed fragments per kilobase of exon per million mapped reads (FPKM), was introduced by Trapnell *et al*. to accommodate paired-end reads [19]; however, this has the same limitation of coupling changes in expression levels among all genes. DESeq computes a scaling factor for a given sample by computing the median of the ratio, for each gene, of its read count over its geometric mean across all samples. It then uses the assumption that most genes are not DE and uses this median of ratios to obtain the scaling factor associated with this sample. Cuffdiff extends this by first performing intra-condition library scaling and then a second scaling between conditions. Cuffdiff also attempts to account for changes in isoform levels explicitly by additional transcript-specific normalization that estimates the abundance of each isoform.

Other normalization procedures attempt to use a subset of stably expressed genes or to normalize within replicated samples to globally adjust library sizes. The trimmed means of M values (TMM) from Robinson and Oshlack [25], which is implemented in edgeR, computes a scaling factor between two experiments by using the weighted average of the subset of genes after excluding genes that exhibit high average read counts and genes that have large differences in expression. Another approach is to sum gene counts up to the upper 25% quantile to normalize library sizes as proposed by Bullard *et al*. [24] and is the default normalization in the baySeq package. The PoissonSeq package uses a goodness-of-fit estimate to define a gene set that is least differentiated between two conditions, which is then used to compute library normalization factors. Quantile normalization ensures that the counts across all samples have the same empirical distribution by sorting the counts from each sample and setting the values to be equal to the quantile mean from all samples [26]. This normalization is widely used in expression arrays and is implemented in the limma package. Recently, a new normalization function termed voom designed specifically for RNA-seq data was added to the limma package. It performs a LOWESS regression to estimate the mean-variance relation and transforms the read counts to the appropriate log form for linear modeling [27].

#### Statistical modeling of gene expression

If sequencing experiments are considered as random samplings of reads from a fixed pool of genes then a natural representation of gene read counts is the Poisson distribution of the form $f\left(n,\lambda \right)=\left(\lambda^{n}e^{-\lambda }\right)/n!$ where *n *is the number of read counts and *λ *is a real number equal to the expected number of reads from transcript fragments. An important property of the Poisson distribution is that the variance is equal to the mean, which equals *λ*. However, in reality the variance of gene expression across multiple biological replicates is larger than its mean expression values [28-30]. To address this over-dispersion problem, methods such as edgeR and DESeq use the related negative binomial distribution (NB) where the relation between the variance *ν *and mean *μ *is defined as *ν *= *μ *+ *αμ*^2 ^where *α *is the dispersion factor.

Estimation of this factor is one of the fundamental differences between the edgeR and DESeq packages. edgeR estimates *α *as a weighted combination of two components: a gene-specific dispersion effect and a common dispersion effect calculated from all genes. DESeq, on the other hand, breaks the variance estimate into a combination of the Poisson estimate (that is, the mean expression of the gene) and a second term that models the biological expression variability. Cuffdiff computes a separate variance model for single-isoform genes and multi-isoform genes. Single-isoform expression variance is computed similarly to DESeq and multi-isoform variance is modeled by a mixture model of negative binomials using the beta distribution parameters as mixture weights. baySeq implements a full Bayesian model of negative binomial distributions in which the prior probability parameters are estimated by numerical sampling from the data. PoissonSeq models the gene counts *N_i_*,*_j _*as a Poisson variable in which the mean *μ_i_*,*_j _*of the distribution is represented by the log-linear relationship log *μ_ij _*= log *d_j _*+ log *β_i _*+ *γ_i_y_j _*where *d_j _*represents the normalized library size, *β_i _*is the expression level of gene *i *and *γ_i _*is the correlation of gene *i *with condition *y_j _*(note that in [8] the subscripts *i *and *j *are samples and genes, respectively). If the expression of gene *i *is not correlated with the sample *j *class (that is, there is no significant difference in gene *i *expression between two conditions) then *γ_i _*is zero.

#### Test for differential expression

The estimation of the parameters for the respective statistical model is followed by the test for differential expression, the calculation of the significance of change in expression of gene *i *between two conditions. Both edgeR and DESeq use a variation of the Fisher exact test adopted for NB distribution; hence, they return exact *P *values computed from the derived probabilities. Cuffdiff uses the test statistics *T *= *E*[log(*y*)]/Var[log(*y*)], where *y *is the ratio of the normalized counts between two conditions, and this ratio approximately follows a normal distribution; hence, a t-test is used to calculate the *P *value for DE. limma uses a moderated t-statistic to compute *P *values in which both the standard error and the degrees of freedom are modified [10]. The standard error is moderated across genes with a shrinkage factor, which effectively borrows information from all genes to improve the inference on any single gene. The degrees of freedom are also adjusted by a term that represents the *a priori *number of degrees of freedom for the model. The baySeq approach estimates two models for every gene, one assuming no differential expression and a second assuming differential expression using the two sample groups. The posterior likelihood of the model of DE, given the observed data, is used to identify differentially expressed genes. In the PoissonSeq method the test for differential expression is simply a test for the significance of the *γ_i _*term (that is, correlation of gene *i *expression with the two conditions), which is evaluated by score statistics. By simulation experiments it was shown that these score statistics follow a chi-squared distribution, which is used to derive *P *values for DE. All methods use standard approaches for multiple hypothesis correction (for example, Benjamini-Hochberg) with the exception of PoissonSeq, which implemented a novel estimation of false discovery rate (FDR) for count data that is based on permutation.

## Results and discussion

### Assessment of normalized counts by sample clustering and log expression correlation

Normalization of read counts is a critical step in the analysis of RNA-seq data that is required to control for the differences in sequencing depths so that gene expression levels can be directly comparable across different samples. In addition, some normalization methods can be used to correct for other effects such as variations in GC content and transcript length [23]. To evaluate the different normalization techniques we performed hierarchical clustering of samples after log_2 _transformation of the normalized count values. We expect that normalization will remove variations that are not due to biological differences and hence the resulting clusters will coincide with biological sources. Indeed, all methods achieved perfect separation between sample types for both the SEQC and the ENCODE datasets suggesting that all normalization methods are able to correct for variable sequencing depths (see Figures S1 and S2 in Additional file 1 and see Materials and methods for a description of samples). The Dunn cluster validity index, which measures the ratios of inter-cluster over intra-cluster distances, indicates a higher cluster separation for the SEQC technical replicate datasets (average Dunn index 3.41) relative to ENCODE biological replicates (average Dunn index 1.00), confirming that biological replicates are more variable than technical replicates (Figure S3 in Additional file 1). The log_2 _distributions of the normalized read counts are similar among most methods with the exception of limmaVoom and Cuffdiff (Figure S4 in Additional file 1), presumably due to the gene-specific normalization approaches by those two methods in contrast to the global scaling that is used by the other methods.

**Figure 1:**
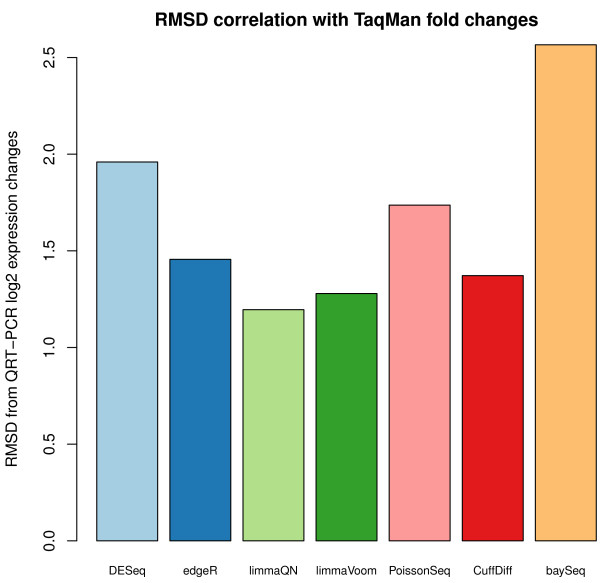
**RMSD correlation between qRT-PCR and RNA-seq log_2 _expression changes computed by each method**. Overall, there is good concordance between log_2 _values derived from the DE methods and the experimental values derived from qRT-PCR measures. Upper quartile normalization implemented in baySeq package is least correlated with qRT-PCR values. DE, differential expression; RMSD, root-mean-square deviation.

Some normalization methods, such as TMM or the goodness-of-fit estimate, are meant to be used in conjunction with a DE testing method and not for direct comparison between samples. As an additional measure of the accuracy of normalization we correlated the log_2 _normalized expression changes reported by each method with log expression changes measured by qRT-PCR, which is only available for the MACQ dataset [31]. Since expression changes are unit-less measures (a ratio of two expression values) we expect the changes to be similar in magnitude and in range regardless of the measurement platform. To assess how accurately the methods matched the PCR data, we used root-mean-square deviation (RMSD) to measure the difference in the reported expression changes to the PCR standard. We found that all methods performed well with an average RMSD accuracy of 1.65 (and Pearson correlation of 0.92) (Figure 1).

### Differential expression analysis

We next evaluated the ability of the various methods to detect differentially expressed genes using both the ERCC and TaqMan data. The ERCC data contains a mixture of spike-in synthetic oligonucleotides that are mixed into samples *A *and *B *at four mixing ratios: 1/2, 2/3, 1 and 4. It is, therefore, possible to test how well the methods correctly identify these ratios. Using the mixing ratio of 1:1 (log ratio = 0) as the true negative set and all others as true positives, we performed a ROC analysis to compare the performance of the various methods in detecting differentially mixed spike-in controls. Overall, all methods performed reasonably well in detecting the truly differentiated spike-in sequences with an average area under the curve (AUC) of 0.78 (Figure S5 in Additional file 1).

**Figure 2:**
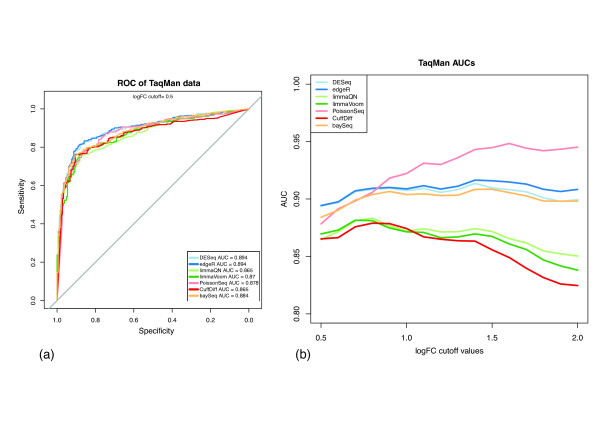
**Differential expression analysis using qRT-PCR validated gene set**. **(a) **ROC analysis was performed using a qRT-PCR log_2 _expression change threshold of 0.5. The results show a slight advantage for DESeq and edgeR in detection accuracy. **(b) **At increasing log_2 _expression ratios (incremented by 0.1), representing a more stringent cutoff for differential expression, the performances of the Cuffdiff and limma methods gradually reduce whereas PoissonSeq performance increases. AUC, area under the curve.

A more comprehensive control group is the set of roughly 1,000 genes whose expression changes were previously measured by qRT-PCR as they span a wider range of expression ratios and represent a sampling of the human transcripts [31]. We performed a ROC analysis using a log_2 _expression change cutoff of 0.5 (1.4 × expression change measured by qRT-PCR) as the threshold for true differentiation. The AUC values at this cutoff indicate comparable performance among all methods with a slight advantage for DESeq and edgeR (Figure 2a). We extended this analysis by measuring AUC at increasing cutoff values of qRT-PCR expression changes, which define sets of differentially expressed genes at increasing stringency (Figure 2b). Here we find a significant performance advantage for negative binomial and Poisson-based approaches with consistent AUC values close to 0.9 or higher in contrast to the Cuffdiff and limma methods, which display decreasing AUC values indicating reduced discrimination power at higher expression change log values.

### Null model evaluation of type I errors

**Figure 3:**
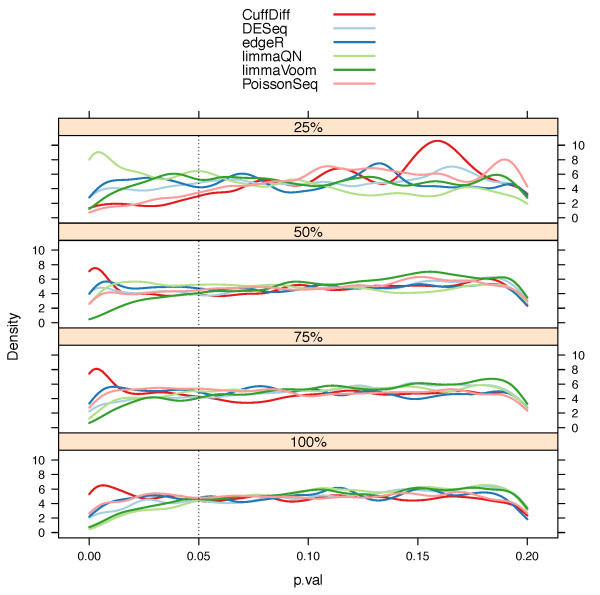
***P *value distributions by gene read count quantiles from null model evaluations**. Null model comparison where differential expression (DE) is evaluated between samples from the same condition is expected to generate a uniform distribution of *P *values. Indeed, the *P *value density plots, stratified by read count quartiles, have a uniform distribution. However, at the common significance range of ≤ 0.05 there is a noticeable increase in *P *value densities in Cuffdiff results indicating larger than expected false DE genes. The smoothing bandwidth was fixed at 0.0065 for all density plots and 25% was the lowest gene read count quartile.

A primary goal for any differential expression algorithm is to minimize type I errors, which are incorrect rejections of the null hypothesis *H*_0_: *μ_i_*,*_A _*= *μ_i_*,*_B_*, where *μ_i_*,*_A_*||*_B _*is the mean expression of gene *i *in condition *A *or *B*, resulting in a false prediction of differential expression (false positive). To test the number of false positive predictions from the null models we performed a series of intra-condition comparisons using the SEQC technical replicate samples from each condition (see Materials and methods). No genes are expected to be differentially expressed in these comparisons and the distribution of *P *values is expected to be uniform since they are derived from the null model. We note that baySeq was excluded from this analysis since it reports posterior probabilities of a model and not *P *values, which does not allow us to control it with the same stringency as other methods. We indeed found that the *P *values for all methods were largely uniform although less so for the lower 25% expressed genes where experimental noise is larger than the expression signal (Figure 3). A noticeable exception was the increase in the *P *values at the lower range (≤0.05) for the Cuffdiff distribution indicating a large number of false positives. A similar observation was noted by Anders *et al*.: Cuffdiff had an inflated number of false positive predictions in their null model comparison [32]. This trend was even more pronounced when the null model comparison was performed without replicated samples (for example, Sample *A *1 vs Sample *A *2, Figure S6 in Additional file 1).

Table 1 summarizes the number of false-positive predictions identified by each method at an adjusted *P *value cutoff (or FDR) of ≤0.05. Null model *P *values were computed from three intra-condition comparisons between replicated samples from the same biological condition (see Materials and methods). In total, 16,287, 16,286, 1,620 and 12,139 *P *values were calculated for genes in the 100%, 75%, 50% and 25% read count quartiles, respectively. Hence, every gene has three reported *P *values from every method representing the three null model comparisons. Note that at the bottom 25% quantile, genes with zero counts were excluded. Although the number of false predictions is below the 5% false discovery rates, the reduced specificity points to inflation of differential expression detection by Cuffdiff. When the comparison was performed with no replicated samples, Cuffdiff's false discovery exceeded 5% where all other methods remained well below this limit.

**Table 1:** Number of false differential expression genes predicted by each method at adjusted *P *values (or false discovery rate) ≤0.05 separated by gene read count quantiles.

Expression quantile	Cuffdiff	DESeq	edgeR	limmaQN	limmaVoom	PoissonSeq	baySeq
100% (high expression)	28	5	3	0	0	7	1
75%	76	6	0	0	0	0	0
50%	84	27	1	2	0	0	0
25% (low expression)	5	9	0	87	0	0	0

Total	193	47	4	89	0	7	1

### Evaluation of genes expressed in only one condition

Almost all RNA-seq experiments include a subset of genes that have no detectable read counts in one of the tested conditions due to very low or lack of expression. In those cases the assessment of differential expression is confounded by the lack of expression signal in one of the tested conditions, which can lead to reduced sensitivity (type II error), or more commonly to *P *values that are inconsistent with the expression levels. Ideally, for this subset of genes the *P *values for differential expression should be monotonically correlated with the signal-to-noise ratios in the expressed condition (*μ*/*σ*, the ratio of the mean over standard deviation) such that higher ratios will be assigned more significant *P *values to reflect the confidence in the expression measurement.

**Figure 4:**
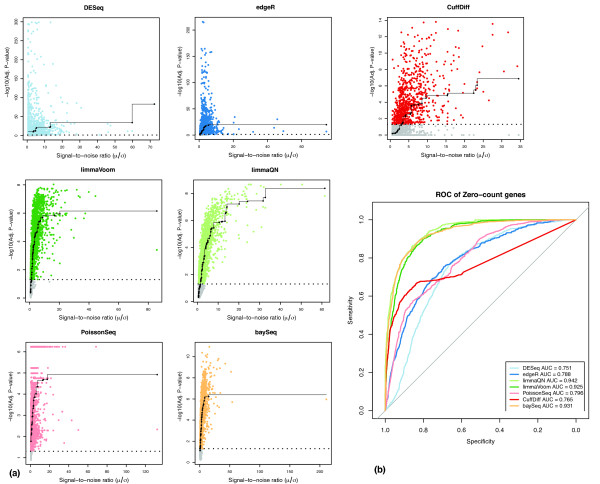
**Comparison of signal-to-noise ratio and differential expression (DE) for genes expressed in only one condition**. **(a) **The correlation between signal-to-noise and -log_10_(*P*) was used to evaluate the accuracy of DE among genes expressed in one condition. A total of 10,272 genes was exclusively expressed in only one of the contrasting conditions in the DE analysis between the three ENCODE datasets. Gray shaded points indicate genes with adjusted *P *value ≥ 0.05, which are typically considered not differentially expressed. The results show that Cuffdiff, edgeR and DESeq do not properly account for variance in measurements as indicated by poor agreement with the isotonic regression line. **(b) **ROC curves for detection of DE at signal-to-noise ratio of ≥3. AUC: area under curve.

We evaluated this correlation using pair-wise comparisons among the three ENCODE datasets. We performed an isotonic regression that models the relation between predictor (signal-to-noise) and response (adjusted *P *value) variables with the added constraint of maintaining a monotonic dependency (that is, if *x_i _*≤ *x_j _*then *f*(*x_i_*) ≤ *f*(*x_j_*)). The results clearly show that the limma and baySeq approaches (and to some extent PoissonSeq) exhibit the desired monotonic behavior between the signal-to-noise and confidence in differential expression as measured by adjusted *P *values whereas DESeq, edgeR and Cuffdiff have poor correlation between these measures (Figure 4). Consistent with the regression analysis, the Kendall-tau rank correlation coefficients also indicate that adjusted *P *values for limma and baySeq are best correlated with signal-to-noise (Figure S7 in Additional file 1). Overall, limma and baySeq had the closest correlation between the two variables demonstrating close to ideal modeling. We postulate that for this subset of genes, DESeq and edgeR methods default to a Poisson model, which implies that the variance is equal to the mean. Hence, the *P *values are well correlated with the mean expression (data not shown) but there is no correction for wide variations in gene counts among replicate libraries.

Incorrect modeling of differential expression in this subset of genes may also result in high levels of false negative or false positive predictions where genes with high signal-to-noise ratios are not identified as differentially expressed or conversely genes with low signal-to-noise are declared to be differentially expressed. Indeed, DESeq and edgeR assign adjusted *P *values of ≤ 0.05 to almost all genes in this dataset regardless of their signal-to-noise values. To measure the sensitivity and specificity we performed a ROC analysis using a signal-to-noise ratio of ≥3 as the classification threshold for differential expression (Figure 4b). The AUC values support the regression results that limma and baySeq had a performance advantage over other methods. Cuffdiff showed significantly reduced specificity relative to other methods as indicated by the large number of false negative genes that have significant signal-to-noise ratios but poor *P *values (gray points below the 1.3 line, that is, adjusted *P *values > 0.05, in Figure 4a). This analysis was repeated with the SEQC datasets with similar results (Figure S8 in Additional file 1).

### Impact of sequencing depth and number of replicate samples on differential expression detection

A common challenge when designing RNA-seq experiment is to maximize the detection power of the study under a limited budget or sample availability. This has raised a number of practical questions. First, what is the desired sequence depth for reliable detection of differential expression and more broadly what is the detection power at a given depth and number of replicates? Second, given a limited sequencing budget, is it preferable to maximize the sequencing depth or increase the number of replicate samples? Finally, what is the impact of different sequencing depths and varying number of replicates on the performances of the DE methods? To address these questions we performed a series of comparisons using combinations of subsets of the sequenced reads and samples. We generated a series of down-sampled libraries where a subset of 50%, 40%, 30%, 20%, 10% and 5% reads were randomly sampled from each library (see Materials and methods). We defined the true set of DE genes as the intersection of the DE genes identified by DESeq, edgeR, limmaVoom and baySeq using the full-size libraries and all five replicates. We then evaluated DESeq, edgeR, limma and PoissonSeq using a decreasing number of replicates and sequence depth, by calculating their: i) sensitivity rates, measured as the fraction of the true set, and ii) false positive (FP) rates, defined as the number of genes identified only by the evaluated algorithm. This analysis was performed on both the SEQC technical replicate samples and the ENCODE biological replicate samples.

**Figure 5:**
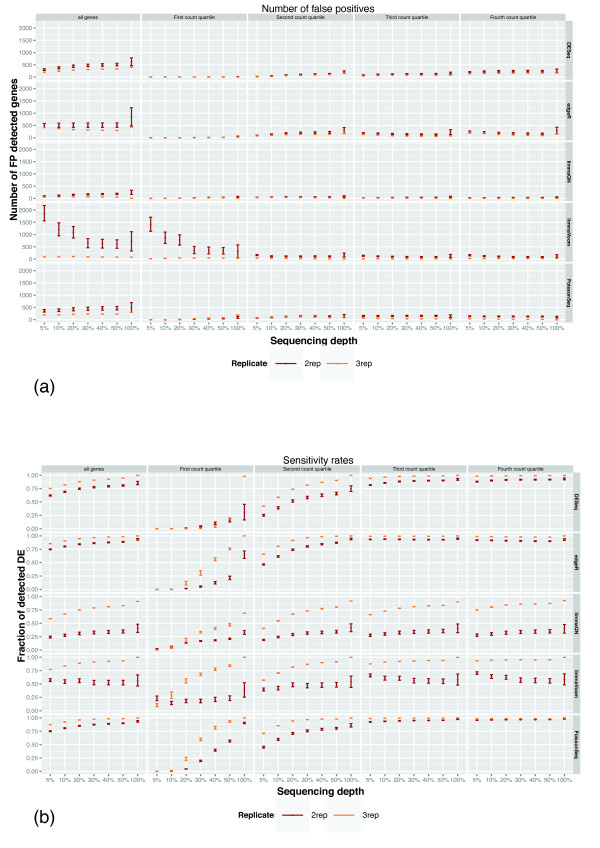
**False positive rates and sensitivity of differential expression (DE) with sequencing depth and number of replicate samples**. Differentially expressed genes in GM12892 vs MCF-7 cell lines were divided into four count quartiles and false positive rate and sensitivity were measured by decreasing sequence counts and changing the number of replicate samples. Points and bars are average and standard deviation, respectively, from five random samples of reads from each library; see Materials and methods for details. **(a) **Number of false positives defined as the number of DE detected genes in GM12892 vs MCF-7 that were only identified by the specific method. **(b) **Sensitivity rates defined as the fraction of true set genes. Note that PoissonSeq's maximum sensitivity is below 1 since it was not included in the definition of the true set. See Figures S9 to S15 in Additional file 1 for similar plots for DE between other cell lines and technical replicates. DE, differential expression; FP, false positive.

As expected, all methods had a smaller number of FPs with increasing number of replications and increased sequencing depths although there are noticeable differences between the methods. limmaQN and edgeR had the lowest rates of FPs whereas DESeq had the highest (Figure 5a and Figures S9 to S15 in Additional file 1). Interestingly, false positive calls among the lowest 25% of expressed genes increased with sequencing depth and number of replicates in contrast to the higher expression quartile where the FP rate reduces when more data is provided. However, the total number of FPs is lowest in the bottom 25% expression indicating that all methods are conservative when predicting DE at low expression ranges.

Sensitivity rates also improve significantly with increased sequencing depth and number of replicates although, here as well, significant variability exists between methods and between expression levels (Figure 5b and Figures S9 to S15 in Additional file 1). Surprisingly, edgeR's sensitivity for the top half of expressed genes decreases with increasing sequence depth (Figure S12 in Additional file 1). This is in contrast to the expected trend that other methods exhibit in which sensitivity improved with increasing number of reads. The most striking effect of sequence depth and number of replicates is apparent in lowly counted genes where sensitivity ranges from <10%, when the comparison is performed with 5% of reads and two replications, to 100% detection when the comparison was performed using the all the reads and all replicates. In contrast, for the highly expressed genes there is little gain in sensitivity with increasing sequencing data or measurements. With most methods, over 90% of differentially expressed genes at the top expression levels are detected with little as two replicates and 5% of the reads.

Taken together these results lead to two conclusions. First, the number of replicate libraries has a greater effect on DE detection accuracy than sequencing depth. This is true for both technical and biological replicates. Second, DE detection of lowly expressed genes is most sensitive to the number of reads and replication whereas there is little benefit to increasing sequencing depths for detecting DE in highly expressed genes.

## Conclusions

In this study we performed a detailed comparative analysis of a number of methods for differential expression analysis from RNA-seq data. For the various methods, our comparison focused on the performance of the normalization, control of false positives, effect of sequencing depth and replication, and on the subset of gene expressed exclusively in one condition. In contrast to other approaches, which rely on simulated data generated by specific statistical distribution or limited experimental datasets [23,33,34], we used the SEQC experimental dataset where a large fraction of the differentially expressed genes were validated by qRT-PCR and biological replicates from three cell lines profiled by the ENCODE project [13]. Overall, no single method emerged as favorable in all comparisons but it is apparent that methods based on negative binomial modeling (DESeq, edgeR, and baySeq) have improved specificity and sensitivities as well as good control of false positive errors with comparable performance. However, methods based on other distributions, such as PoissonSeq and limma, compared favorably and have improved modeling of genes expressed in one condition. On the other hand, Cuffdiff has reduced sensitivity and specificity as measured by ROC analysis as well as the significant number of false positives in the null model test. We postulate that this is related to its normalization procedure, which attempts to account for both alternative isoform expression and length of transcripts. Table 2 summarizes the comparison results in addition to a number of additional quality measures, which were not directly evaluated in this study.

**Table 2:** Comparison of methods.

Evaluation	Cuffdiff	DESeq	edgeR	limmaVoom	PoissonSeq	baySeq
Normalization and clustering	All methods performed equally well					
DE detection accuracy measured by AUC at increasing qRT-PCR cutoff	Decreasing	Consistent	Consistent	Decreasing	Increases up to log expression change ≤ 2.0	Consistent
Null model type I error	High number of FPs	Low number of FPs	Low number of FPs	Low Number of FPs	Low number of FPs	Low number of FPs
Signal-to-noise vs *P *value correlation for genes detected in one condition	Poor	Poor	Poor	Good	Moderate	Good
Support for multi-factored experiments	No	Yes	Yes	Yes	No	No
Support DE detection without replicated samples	Yes	Yes	Yes	No	Yes	No
Detection of differential isoforms	Yes	No	No	No	No	No
Runtime for experiments with three to five replicates on a 12 dual-core 3.33 GHz, 100 G RAM server	Hours	Minutes	Minutes	Minutes	Seconds	Hours

AUC, area under curve; DE, differential expression; FP, false positive.

Surprisingly, the limma package, which was developed and optimized for expression array analysis, had comparable, and by some measures improved, performance for both normalization versions tested relative to the other models, which were tailored for RNA-seq analysis. Furthermore, the difference between quantile normalization or the RNA-seq specific voom function in limma was evident in the number of false DE genes in the null model and in the sensitivity to the sequencing depth and number of replicated samples. limma models the data as a normal distribution, which is a reasonable assumption for array intensities but perhaps counterintuitive for count data since it models discrete data with a continuous distribution. However, it is plausible that in the limit of large counts it is more important to model the variance accurately than the discreteness. This study demonstrates that for datasets with a large number of genes (or tags), the limma package is well suited for detecting DE genes and that modeling gene count data as a log normal distribution, with the appropriate pseudo counts, is a reasonable approximation.

The results from sequencing depth and replication analysis demonstrate conclusively that the number of sample replicates is the most significant factor in accurate identification of DE genes [33]. This is not surprising considering that the focus of most methods is to model the variability in gene expression measurements and therefore increasing the number of replicates adds power to this estimate. Since the squared signal-to-noise improves with increased mean expression [35], DE among the highly expressed genes is easily detected even with low sequencing depth and few sample replicates. From a practical point of view, studies focused on detecting DE among lowly expressed genes will benefit significantly from an increased number of replicates. Many additional factors that directly impact the detection of differential expression were not considered in this study such as choice of alignment algorithm, derivation of gene counts, multi-factored studies, detection of alternative transcripts and choice of sequencing platform. Cuffdiff, for example, incorporates differential isoform detection, which is not supported by the simple gene counting methods evaluated here. It is also important to note that the evaluated methods may not be applicable to all types of RNA-seq data. For example, small RNA sequencing is not always amenable to quantile normalization as performed in this study (data not shown). Similarly, RNA-seq data from cross-linking and immunoprecipitation (CLIP) or RIP-seq from RNA-binding proteins are fundamentally different in nature from typical transcriptome profiling and therefore require specialized models. Finally, the field of high-throughput sequencing is rapidly evolving with new technologies being continuously introduced. These add additional elements of variability to the measurements and will require specific consideration [36].

The emergence of RNA-seq as the method of choice for transcriptional profiling has motivated the development of a growing number of algorithms for normalization and analysis. This comparative study is the first exhaustive comparison of the widely used DE methods on experimental data. It provides important guidelines for evaluating RNA-seq analysis methods and points the direction for future improvements.

## Materials and methods

### Datasets

In this study, we used samples from two sources that were part of the SEQC study, each generated from a mixture of biological sources and a set of synthetic RNAs from the External RNA Control Consortium (ERCC) at known concentrations. The samples from group *A *contain the Strategene Universal Human Reference RNA (UHRR), which is composed of total RNA from ten human cell lines, with 2% by volume of ERCC mix 1. The second group of samples *B *contains Ambion's Human Brain Reference RNA (HBRR) with 2% by volume of ERCC mix 2. The ERCC spike-in control is a mixture of 92 synthetic polyadenylated oligonucleotides, 250 to 2,000 nucleotides long, which are meant to resemble human transcripts. The two ERCC mixtures in groups *A *and *B *contain different concentrations of four subgroups of the synthetic spike-ins such that the log expression change is predefined and can be used to benchmark DE performance (see the Methods section in main SEQC publication). Four replicate libraries from groups *A *and *B *were prepared by a single technician and a fifth sample was prepared by Illumina for a total of ten libraries. All libraries were sequenced as paired-end 100 bases in the Epigenomics Core facility at Weill Cornell Medical College with a full block design on two flow cells on a single HiSeq2000 instrument (GEO accession GSE49712). We note that these samples are considered technical replicates and therefore represent an idealized scenario of minimal variation.

ENCODE Biological replicate datasets were generated by the ENCODE project [13] and the fastq files were downloaded [14]. We used replicate libraries from human cell lines GM12892 (three replicates), H1-hESC (four replicates) and MCF-7 (three replicates) sequenced as 75 paired-ends at the CalTech center. To determine whether the ENCODE data adequately represents the variability seen in biological samples we plotted the mean of the normalized counts against the variance for the three cell lines (Figure S16 in Additional file 1). The results show that the variance does increase more rapidly than the mean indicating that the ENCODE data is indeed over-dispersed and is a good model for the variability seen in biological replicates.

### Sequence alignment and gene counts

All sequenced libraries were mapped to the human genome (hg19) using TopHat(v.2.0.3) [5] with the following parameters: '-r 70 --mate-std-dec 90'. A custom GTF file that includes both RefSeq information (from the UCSC genome browser) and the ERCC transcript information was used (--GTF $SEQCLB/hg19_150_ERCC.gtf) along with the transcriptome index option (--transcriptome-index $SEQCLIB/hg19_150_ERCC). Genes shorter than 150 bp were excluded from this GTF file. HTSeq (v.0.5.3p3) [15] was used to generate the count matrix with the following parameters: 'htseq-count -m intersection-strict -s no' with the same GTF file used for the alignment step ($SEQCLIB/hg19_150_ERCC.gtf).

### Normalization and differential expression

With the exception of Cuffdiff, all differential expression analysis was performed using the same gene count matrix output from HTSeq. Analysis followed the procedures and steps described in the package documentation and unless stated otherwise default parameters were used in all function calls. Adjusted *P *values for multiple hypothesis corrections were used as calculated by the methods. The following are the details for each package used in this study:

• DESeq (v.1.10.1): The dispersion estimate call to estimateDispersions had parameters: 'method="per-condition"' and 'fitType="local"' and for null model evaluation with no replicates 'method="blind"', 'fitType="local"' and 'sharingMode="fit-only"'.

• edgeR (v.3.0.2): In the null model comparison with no replicates the common.dispersion value was set to 0.4 as suggested by the documentation.

• PoissonSeq (v.1.1.2): No minimum expression mean was applied and the number of permutations was 500.

• baySeq (v.1.12.0): Sequence length correction was added to the normalization as suggested in the documentation. Negative binomial parameter estimation was performed using getPriors.NB using quasi-likelihood estimation. Note that baySeq reports posterior probabilities for differences between two models and not *P *values.

• limma(v.3.14.1) Analysis was performed in two modes, which have different normalization procedures. Quantile normalization was performed on the log_2 _transformed gene counts (with the addition of 1 to avoid a log of 0) by normalizeBetweenArrays function (known as limmaQN). In the second mode, counts were normalized using the voom function where library sizes were scaled by edgeR normalization factors and the mean-variance trend was calculated using LOWESS regression (known as limmaVoom). Note that limma does not allow contrasting libraries with no replication and therefore limma was excluded from the single library comparisons.

• cuffdiff (v.2.0.0 (3365)) with the options: '--no-update-check --emit-count-tables' and GTF file $SEQCLIB/hg19_150_ERCC.gtf.

For each method, comparisons were performed between the five replicates from sample type *A *with the five replicates from type *B*. In the null model comparison two models were tested, with replication and without replication. In the replication model, replicates from the same samples were contrasted: {*A*1, *A*2} vs {*A*3, *A*4}, {*A*1, *A*2} vs {*A*3, *A*4, *A*5} and {*B*1, *B*2} vs {*B*3, *B*4}. Comparisons without replication were performed between the following samples: *A*1 vs *A*2, *A*3 vs *A*4, *B*1 vs *B*2 and *B*3 vs *B*4.

### Sample clustering

Normalized counts were log_2 _transformed after addition of pseudo counts. For counts produced by HTSeq the pseudo counts were set to the smallest non-zero gene count in each library and for FPKM data the pseudo count was set to 0.001. Clustering was performed using the R hclust function with the Euclidean distance measure.

### Random sampling and sequencing depth

To assess the effect of a reduced sequencing depth, we used DownsampleSam, a function from Picard [16] that randomly samples read pairs from a SAM file using a uniform probability. We generated a first set of reduced coverage depth samples by subsampling every sequence library with a probability of *p*_1 _= 0.5 for retaining each read. We then subsampled the resulting files with a probability *p*_2 _= 0.8. Therefore, we generated a set that subsampled the original files with a probability *p*_1 _× *p*_2 _= 0.4 representing 40% sequencing depth. We continued this subsampling cascade, ultimately generating six sets of files with 0.5, 0.4, 0.3, 0.2, 0.1 and 0.05 of the reads sampled from the original files. We then repeated the operation five times, generating five random datasets for each fraction value.

For each subsampled fraction, we used the five independent samplings to compute differential expression between every combination of subsets of samples (for example, all groups of two samples from condition *A *compared to all groups of two samples from condition *B*). We evaluated the DE using DESeq, edgeR, PoissonSeq and limma using the two described modes.

### Source code

The source code and data files are available online [37].

## List of abbreviations used

AUC: area under the curve; bp: base pair; CLIP: cross-linking and immunoprecipitation; DE: differential expression; ERCC: External RNA Control Consortium; FDR: false discovery rate; FP: false positive; FPKM: fragments per kilobase of exon per million mapped reads; HBRR: Human Brain Reference RNA; NB: negative binomial; RMSD: root-mean-square deviation; RPKM: reads per kilobase per million reads; SEQC: Sequencing Quality Control; TMM: trimmed means of M values; UHRR: Universal Human Reference RNA.

## Competing interests

The authors declare that they have no competing interests.

## Authors' contributions

DB, FR, RK, YL, MP and AK performed the analysis. CEM and PZ performed the sequencing experiments. DB, NDS and RK led the study and DB, FR, NDS and RK wrote the manuscript.

## Supplementary Material

Additional file 1 **Supplementary figures**. All the supplementary figures referenced in the main text. 1 Hierarchical clustering of the SEQC libraries from sample A and B . . . . . . . . . . 3. 2 Hierarchical clustering of the ENCODE samples .................... 4. 3 Dunn clustering validity index............................... 5. 4 Normalized read counts .................................. 6. 5 ROC analysis of ERCC spike-in controls.........................7. 6 Null model p-values distribution without replicate samples . . . . . . . . . . . . . . . 8. 7 Evaluating monotonic correlation between signal-to-noise and p-values in genes expressed in only one condition ............................... 9. 8 Correlation of signal-to-noise ratio and DE p-values from SEQC data set . . . . . . . 10. 9 Methods performances with reduced sequencing depth and number of replicates for detecting DE between GM12892 and H1-hESC............... 11. 10 Methods performances with reduced sequencing depth and number of replicates for detecting DE between H1-hESC and MCF-7.................. 12. 11 Impact of sequencing depth and number of replicate samples on DE detection by DESeq using SEQC data.................................. 13. 12 Impact of sequencing depth and number of replicate samples on DE detection by edger using SEQC data.................................. 14. 13 Impact of sequencing depth and number of replicate samples on DE detection by limmaQN using SEQC data................................ 15. 14 Impact of sequencing depth and number of replicate samples on DE detection by limmaVoom using SEQC data............................. 16. 15 Impact of sequencing depth and number of replicate samples on DE detection by PoissonSeq using SEQC data ............................. 17. 16 Over-dispersion of the ENCODE dataset ........................ 18}. Click here for file
