# Erratum to: Comprehensive evaluation of differential gene expression analysis methods for RNA-seq data

**DOI:** 10.1186/s13059-015-0813-z

**Published:** 2015-11-23

**Authors:** Franck Rapaport, Raya Khanin, Yupu Liang, Mono Pirun, Azra Krek, Paul Zumbo, Christopher E. Mason, Nicholas D. Socci, Doron Betel

**Affiliations:** Bioinformatics Core, Memorial Sloan-Kettering Cancer Center, New York, NY 10065 USA; Department of Physiology and Biophysics, Weill Cornell Medical College, New York, NY 10021 USA; Institute for Computational Biomedicine, Weill Cornell Medical College, New York, NY 10021 USA; Division of Hematology/Oncology, Department of Medicine, Weill Cornell Medical College, New York, NY 10021 USA

We previously published a report on the comprehensive evaluation of RNA-seq differential analysis (DE) methods [1] where we compared a number of popular DE tools using a variety of different criteria. Since publication we received valuable feedback and suggestions from the community including from the authors of the algorithms. Here we report on two errors that came to our attention following publication.

1. Soon after publication we were notified about a discrepancy in table 2. The correction was posted as comment to the main article and now included as erratum. In the last row of table 2 called "Runtime for experiments with 3-5 replicates…" the values for edgeR and limmaVoom should be Seconds not Minutes.

For completeness, the following table is the runtime performance (in seconds) of six DE analysis methods, comparing the 5 replicates from groups A and B from the SEQC data, as measured on Red Hat Enterprise Linux Server release 5.4, with 12 dual cores Intel Xeon 3.33GHz, and 100G RAM.

**Table:** 

	DESeq	edgeR	PoissonSeq	limmaVoom	baySeq
user.self	410.292	7.380	12.893	4.897	20.772
sys.self	0.356	0.004	0.153	0.011	0.166
elapsed	413.751	7.904	13.343	4.911	2028.279

2. Zhou and Robinson performed a follow up analysis to our manuscript where they reanalyzed DE when genes are expressed in one condition (see correspondence to Rapaport et al.). They identified a coding error in the calculation of edgeR signal to noise values due to incorrect normalization of edgeR count values. Library size values were not used to scale gene counts. This coding error was fixed and a corrected version is deposited in the source code repository available at: http://bitbucket.org/soccin/seqc.

We redid the analysis for evaluation of genes expressed in only one condition and the main conclusions remain unchanged. The corrected version of Figure 4 in the main manuscript is presented here as Fig. 1.

**Fig. 1:**
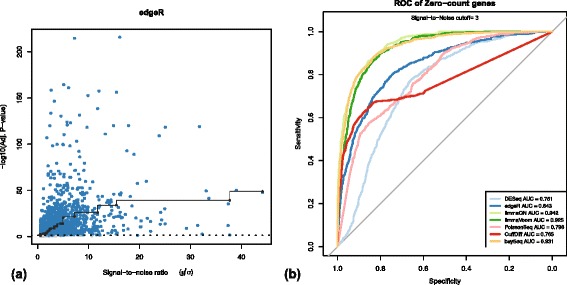
**a** edgeR correlation between signal-to-noise and –log10(p-values) using the corrected normalization of gene counts. **b** ROC analysis of curves for detection of DE at signal-to-noise ratio of ≥3. Note that edgeR AUC has improved from 0.788 to 0.843 following the correction of normalization. All other panels in the original figure remain unchanged

We note that our reanalysis is based on the exact same procedure used in the original publication. However, Zhou and Robinson introduce a different version of this analysis where the ROC analysis is based on a common set of genes. Second, most packages used in our original publication have since been updated and in some cases the algorithms revised substantially. Therefore, it is possible that a similar comparison with the latest versions of the packages may result in different conclusions.
