# Supplementary material for: Comprehensive evaluation of differential gene expression analysis methods for RNA-seq data
Source: Genome Biol. 2013 Sep 10;14(9):R95. doi: 10.1186/gb-2013-14-9-r95 (PMC4054597; doi:10.1186/gb-2013-14-9-r95)
Supplement: Additional file 1 — Supplementary figures. All the supplementary figures referenced in the main text. 1 Hierarchical clustering of the SEQC libraries from sample A and B . . . . . . . . . . 3. 2 Hierarchical clustering of the ENCODE samples .................... 4. 3 Dunn clustering validity index............................... 5. 4 Normalized read counts .................................. 6. 5 ROC analysis of ERCC spike-in controls.........................7. 6 Null model p-values distribution without replicate samples . . . . . . . . . . . . . . . 8. 7 Evaluating monotonic correlation between signal-to-noise and p-values in genes expressed in only one condition ............................... 9. 8 Correlation of signal-to-noise ratio and DE p-values from SEQC data set . . . . . . . 10. 9 Methods performances with reduced sequencing depth and number of replicates for detecting DE between GM12892 and H1-hESC............... 11. 10 Methods performances with reduced sequencing depth and number of replicates for detecting DE between H1-hESC and MCF-7.................. 12. 11 Impact of sequencing depth and number of replicate samples on DE detection by DESeq using SEQC data.................................. 13. 12 Impact of sequencing depth and number of replicate samples on DE detection by edger using SEQC data.................................. 14. 13 Impact of sequencing depth and number of replicate samples on DE detection by limmaQN using SEQC data................................ 15. 14 Impact of sequencing depth and number of replicate samples on DE detection by limmaVoom using SEQC data............................. 16. 15 Impact of sequencing depth and number of replicate samples on DE detection by PoissonSeq using SEQC data ............................. 17. 16 Over-dispersion of the ENCODE dataset ........................ 18}. [file gb-2013-14-9-r95-S1.PDF]

Supplementary Material: Comprehensive evaluation of differential gene expression analysis methods for RNA-seq data

Franck Rapaport [1], Raya Khanin [1], Yupu Liang [1], Mono Pirun[1], Azra Krek [1], Paul Zumbo [2,4], Christopher E. Mason [2,4], Nicholas D. Socci [1], Doron Betel [3,4]

[1]Bioinformatics Core, Memorial Sloan-Kettering Cancer Center, New York
[2]Department of Physiology and Biophysics, Weill Cornell Medical College, New York
[3] Division of Hematology/Oncology, Department of Medicine, Weill Cornell Medical College, New York
[4] Institute for Computational Biomedicine, Weill Cornell Medical College, New York

July 5, 2013

List of Figures

1 Hierarchical clustering of the SEQC libraries from sample A and B 3
2 Hierarchical clustering of the ENCODE samples 4
3 Dunn clustering validity index 5
4 Normalized read counts 6
5 ROC analysis of ERCC spike-in controls 7
6 Null model p-values distribution without replicate samples 8
7 Evaluating monotonic correlation between signal-to-noise and p-values in genes expressed in only one condition 9
8 Correlation of signal-to-noise ratio and DE p-values from SEQC data set 10
9 Methods performances with reduced sequencing depth and number of replicates for detecting DE between GM12892 and H1-hESC 11
10 Methods performances with reduced sequencing depth and number of replicates for detecting DE between H1-hESC and MCF-7 12
11 Impact of sequencing depth and number of replicate samples on DE detection by DESeq using SEQC data 13
12 Impact of sequencing depth and number of replicate samples on DE detection by edgeR using SEQC data 14
13 Impact of sequencing depth and number of replicate samples on DE detection by limmaQN using SEQC data 15
14 Impact of sequencing depth and number of replicate samples on DE detection by limmaVoom using SEQC data 16
15 Impact of sequencing depth and number of replicate samples on DE detection by PoissonSeq using SEQC data 17

16 Over-dispersion of the ENCODE data set 18

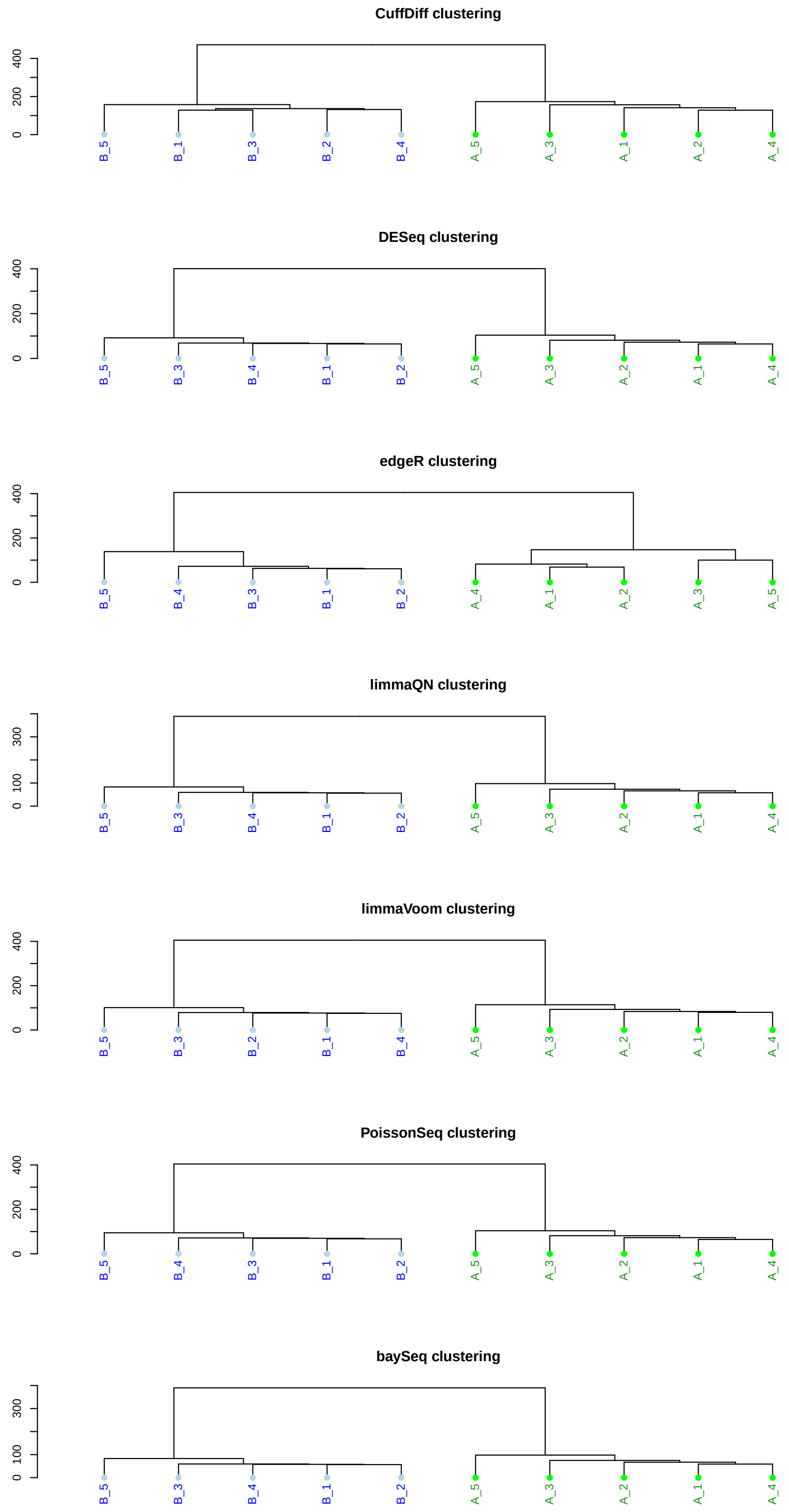

Figure S 1: **Sample clusters.** Hierarchical clustering of the SEQC libraries from sample **A** and **B**. Read counts were normalized by each method and log2 transformed. All methods achieved perfect separation of libraries by sample type.

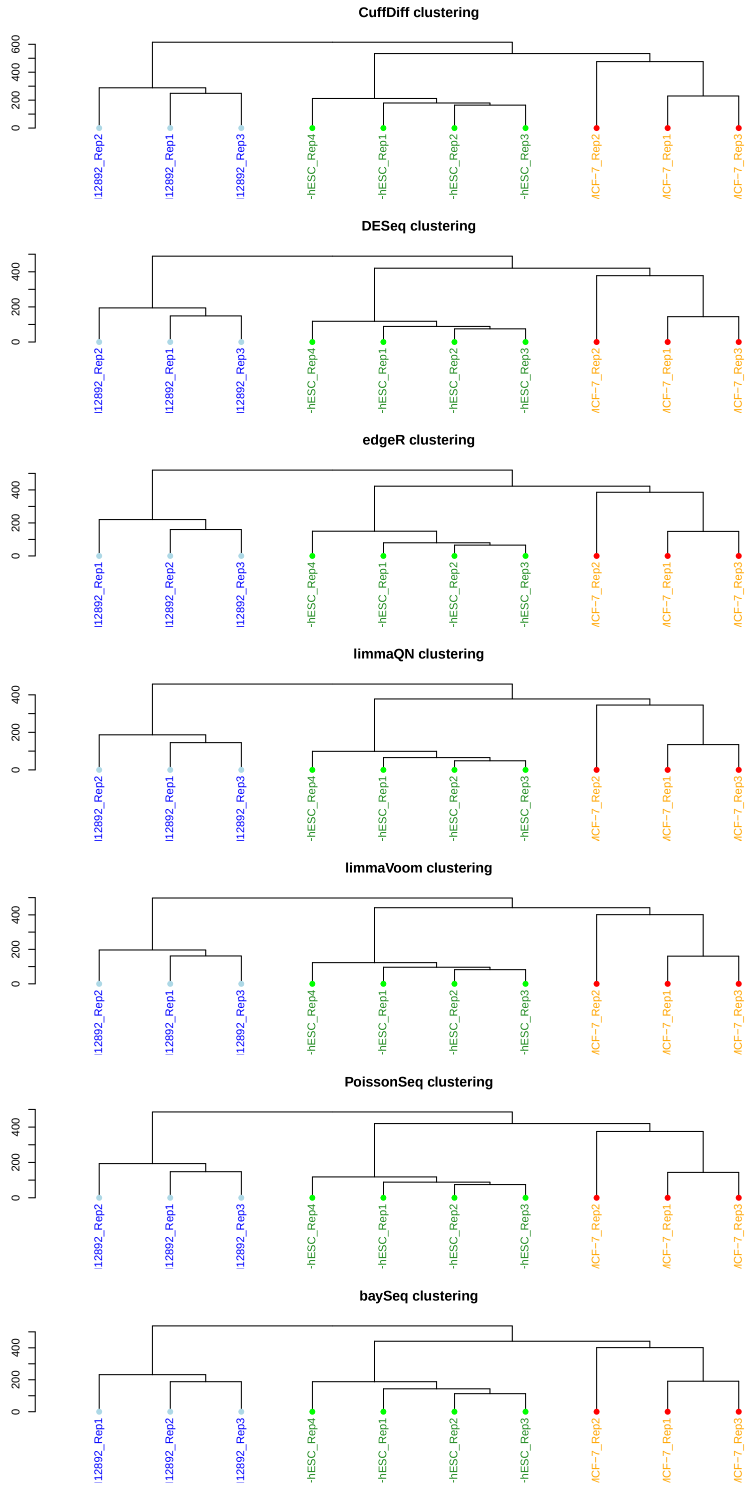

Figure S 2: **Sample clusters.** Hierarchical clustering of the ENCODE libraries from **GM12892**, **H1-hESC** and **MCF-7**. clustering was performed similarly as Figure S1.

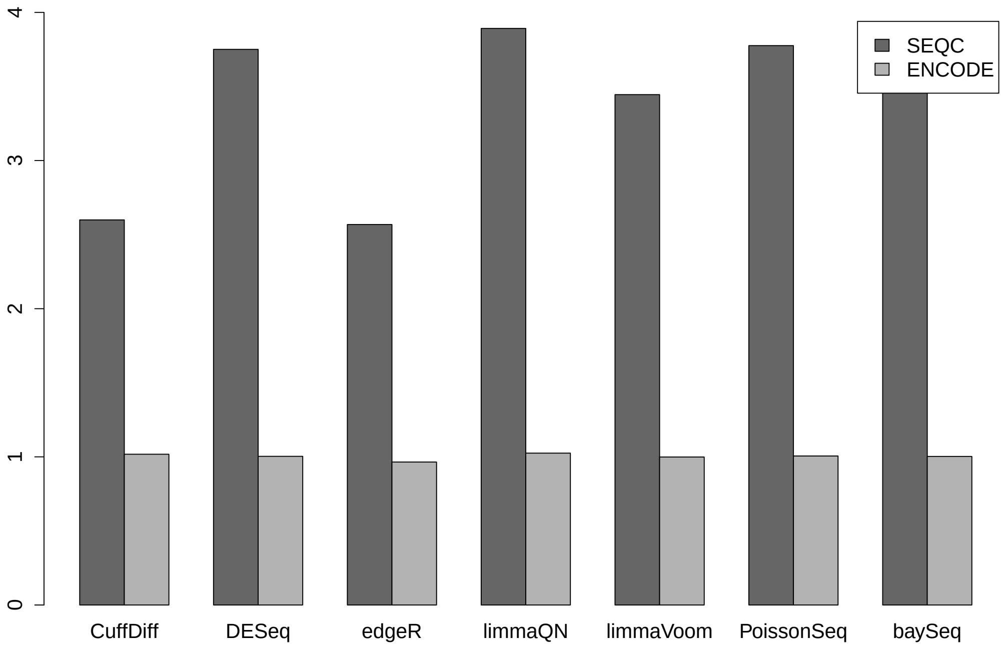

Figure S 3: **Dunn clustering validity index**. Dunn index was used to evaluate clustering of normalized gene count data from both SEQC and ENOCDE data. Higher values indicate better clustering with closer distances within clusters than between clusters.

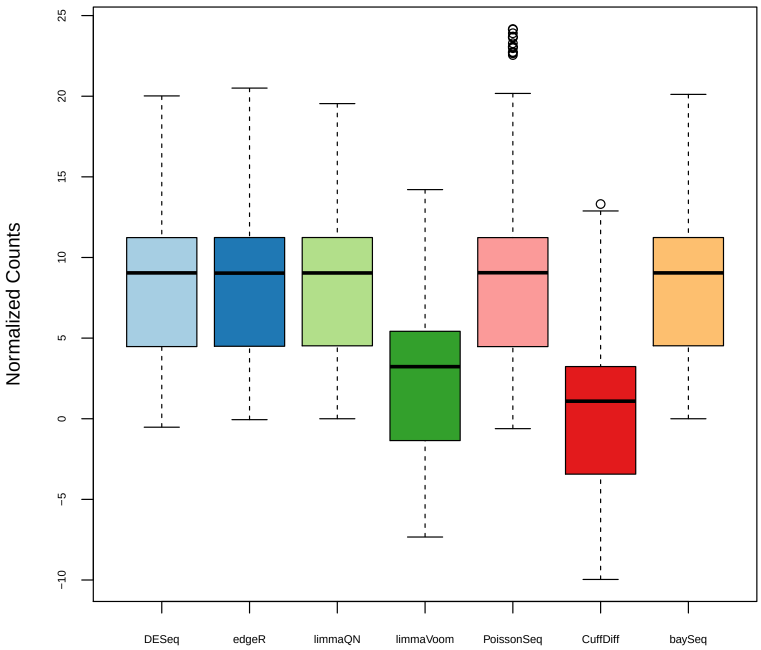

Figure S 4: **Normalized read counts.** Boxplots of the normalized read counts distributions from all 10 samples. Normalization based on a single scaling factor result in similar count distributions while gene-specific normalization such as limmaVoom or Cuffdiff are markedly different.

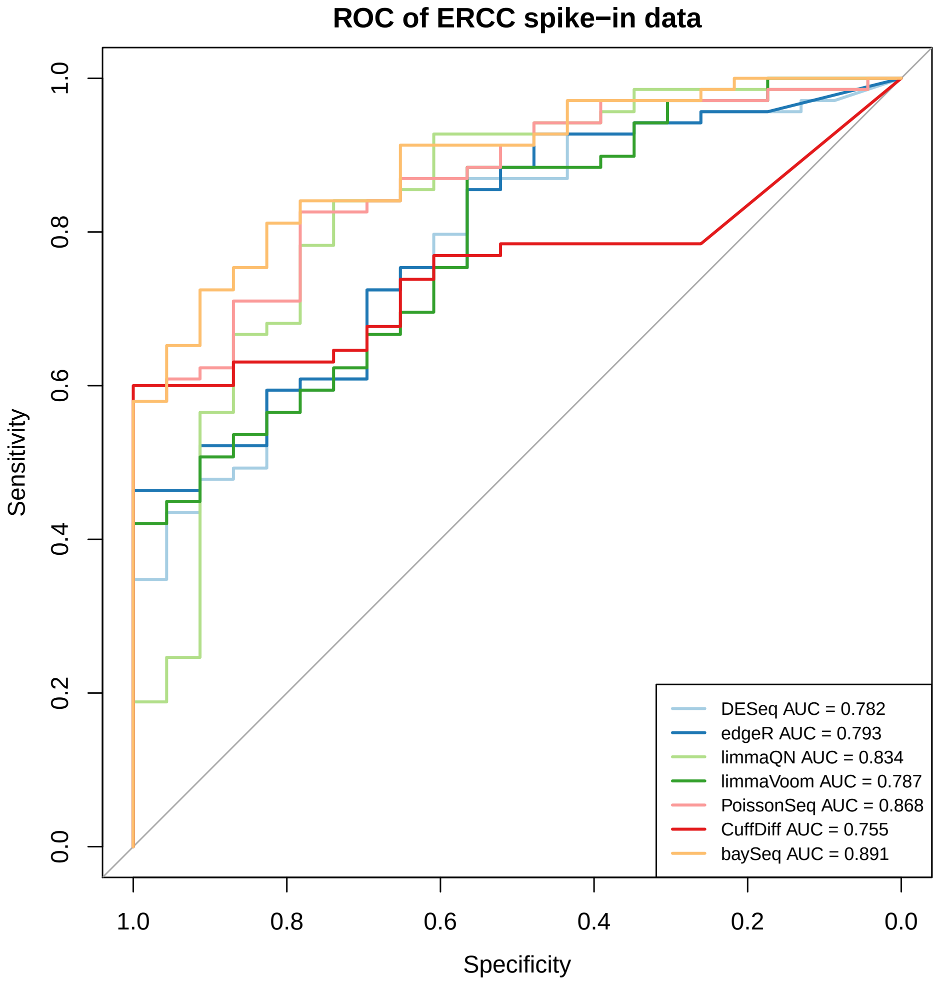

Figure S 5: **ROC analysis of ERCC spike-in controls.** ERCC control oligonucleotides were divided into four groups with different mixing ratios between samples **A** and **B** (1:1, 4:1, 1:2 and 2:3). In this ROC analysis the 1:1 mix are the set of undifferentiated controls (true negatives) and all others are differentiated(true positives). AUC = area under the curve.

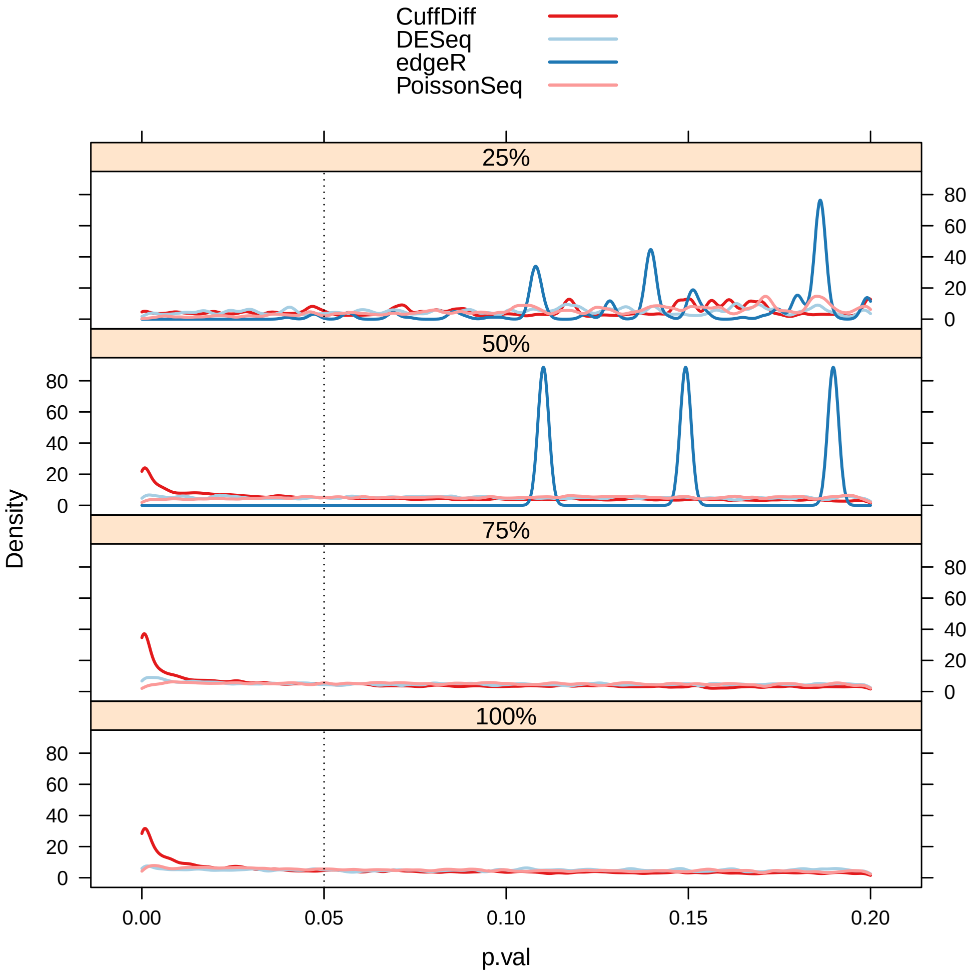

Figure S 6: **Null model p-values distribution without replicate samples.** Density plots of null model p-values generated from comparisons without replication (i.e. comparison between two samples A_i vs. A_j or B_i vs. B_j). Results were separated based on four read counts quartiles. In most cases p-value densities are uniform as expected from null model comparison however, Cuffdiff p-values are significantly enriched in the lower range (≤ 0.05) indicating a larger number than expected of false positive prediction of differential expression. Note that limma does not allow contrasting conditions with no replicated samples and therefore was excluded from this analysis.

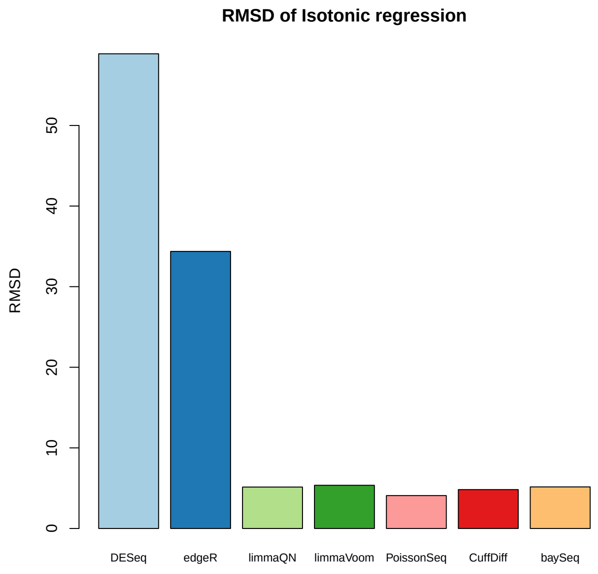

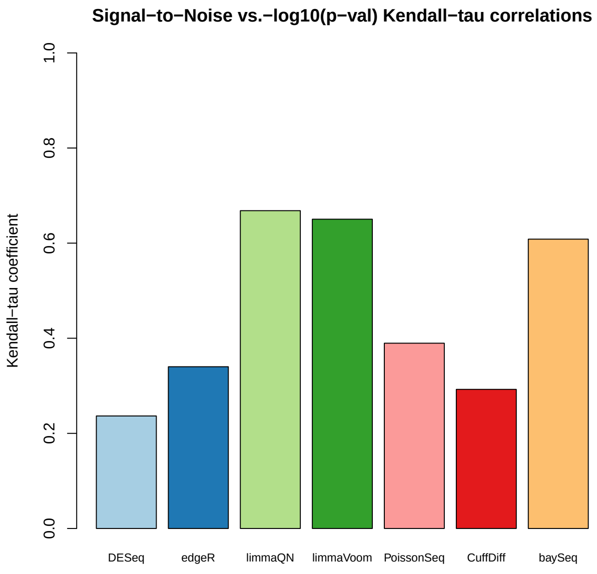

Figure S 7: **Evaluating monotonic correlation between signal-to-noise and p-values in genes expressed in only one condition**.(**a**) Isotonic regression modeled the monotonic correlation between signal-to-noise and p-values for the subset of genes with counts in only one of the two conditions. RMSD between observed and predicted values shows that DESeq and edgeR deviated significantly from the desired monotonic relationship suggesting that for this subset of genes the methods do not model properly the variations in gene count measurements. (**b**) Kendall-tau rank correlation between signal-to-noise in the expressed condition and adjusted p-values.

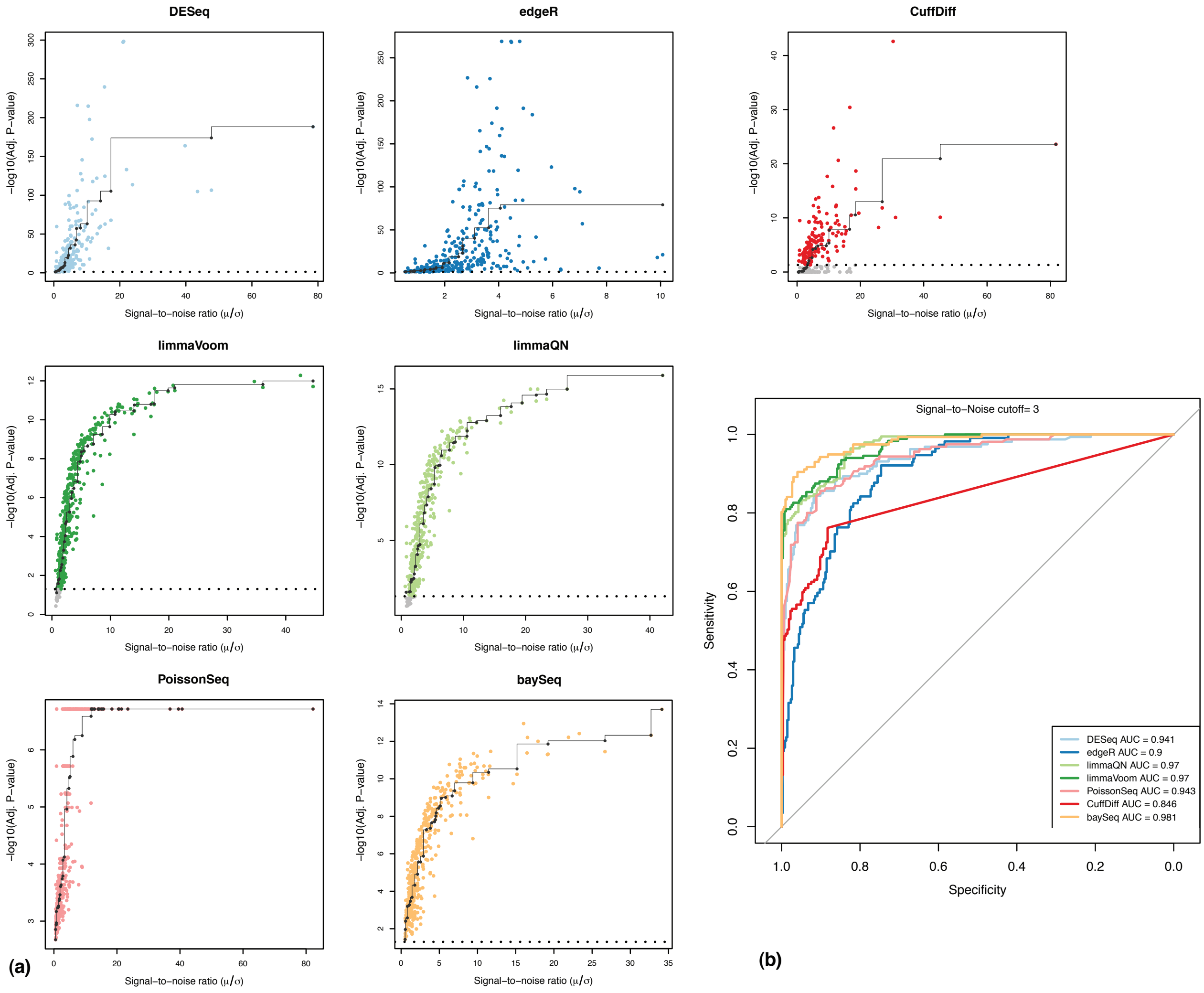

Figure S 8: **Comparison of signal-to-noise ratios and DE p-values using SEQC data.** Similar analysis as Figure 4 in manuscript using SEQC data set.

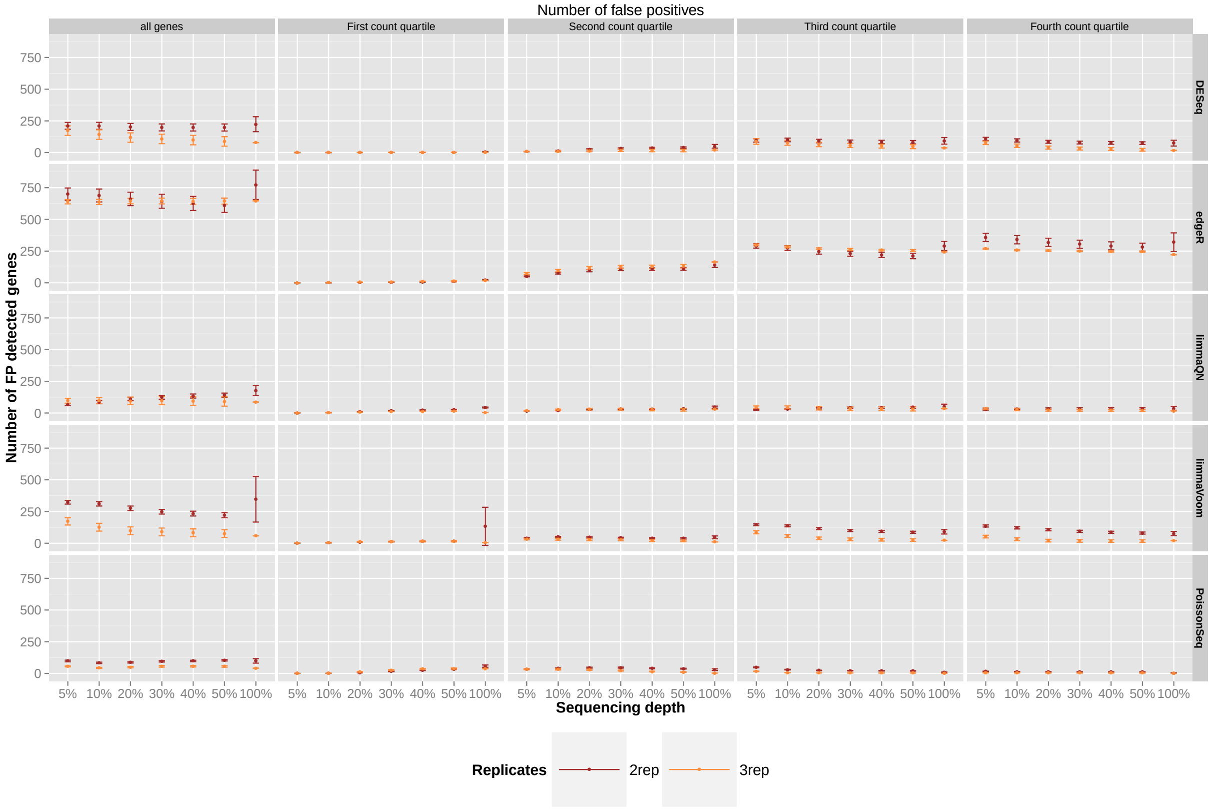

(a) False positive rates defined as the number of incorrectly identified DE genes

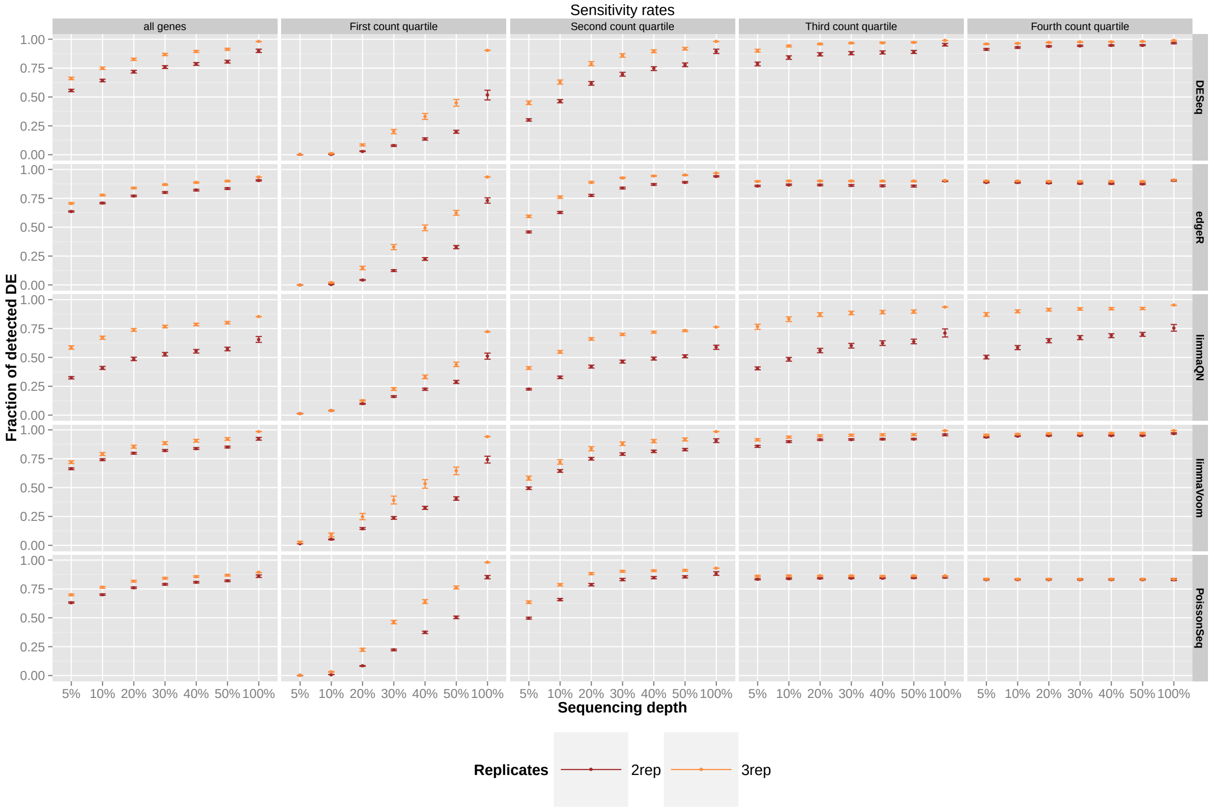

(b) Sensitivity defined as the fraction of DE genes identified from the true set

Figure S 9: Analysis of methods performance with varying sequencing depth and number of replicates for detecting DE between GM12892 and H1-hESC.

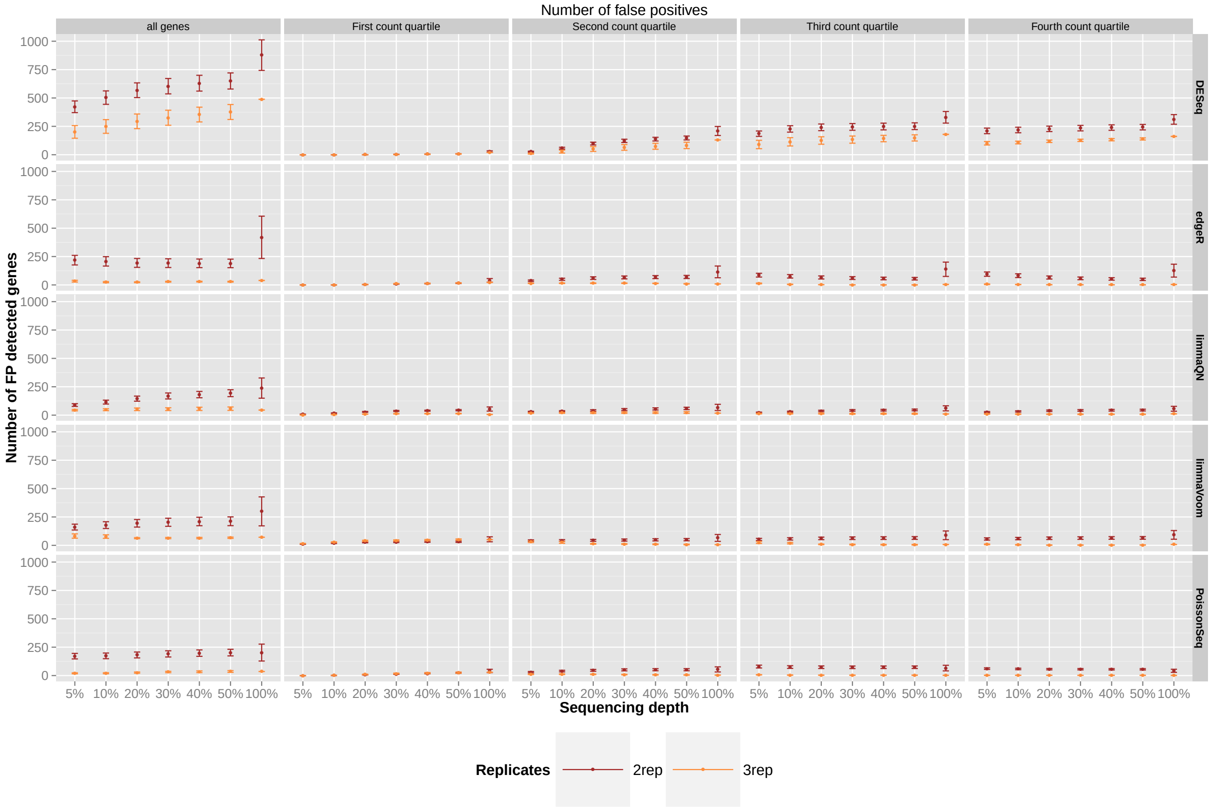

(a) False positive rates defined as the number of incorrectly identified DE genes

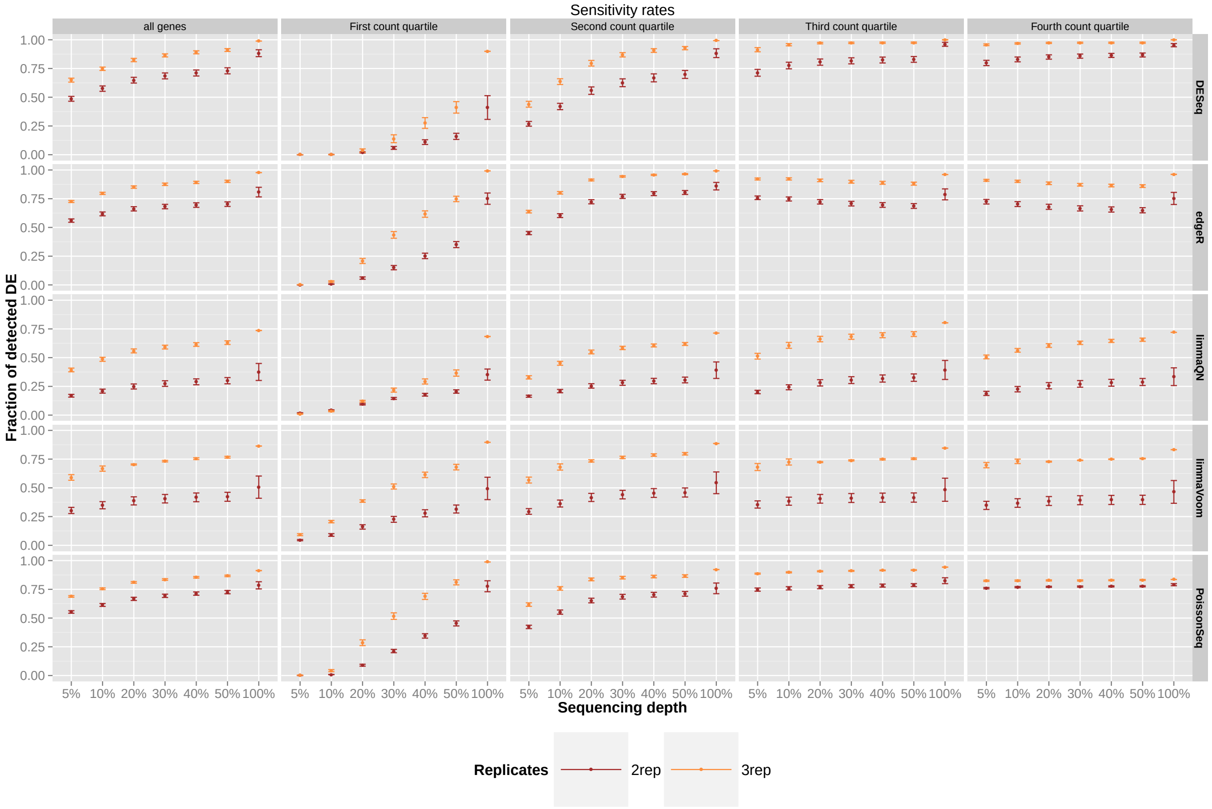

(b) Sensitivity defined as the fraction of DE genes identified from the true set

Figure S 10: Analysis of methods performance with varying sequencing depth and number of replicates for detecting DE between H1-hESC and MCF-7.

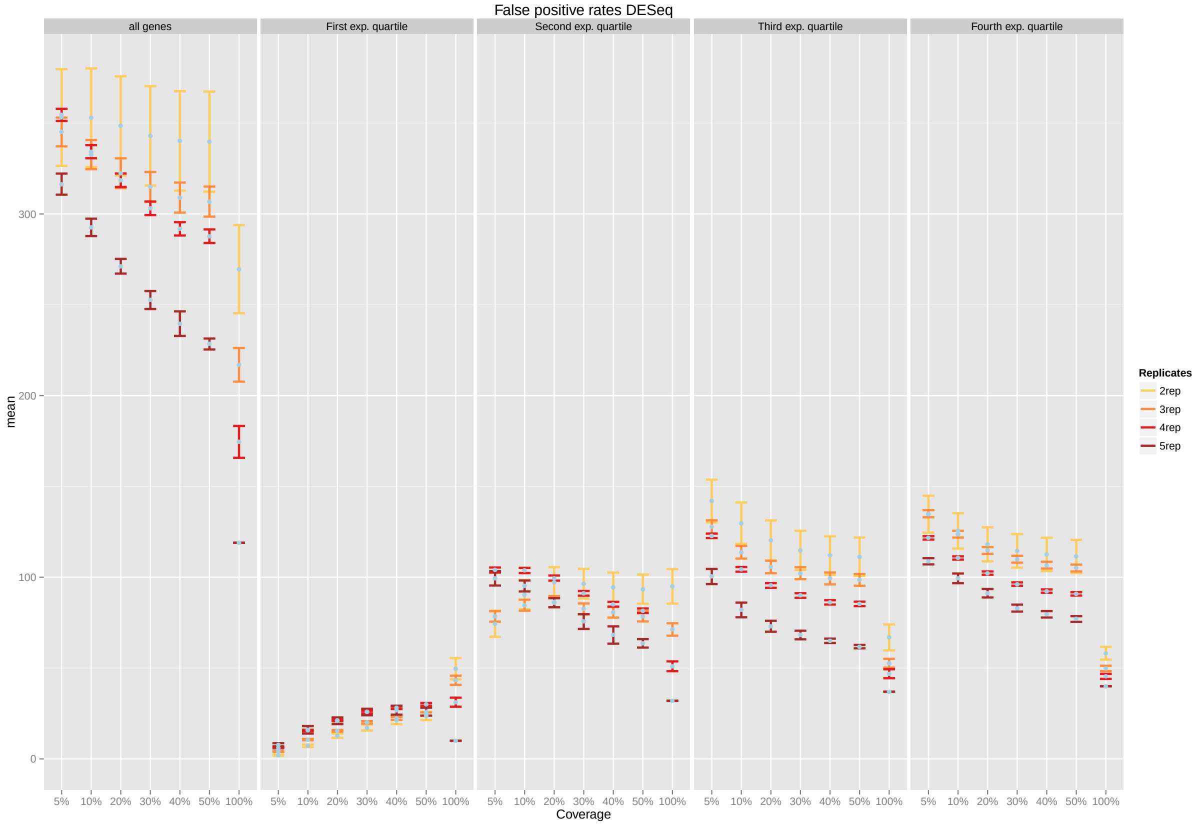

(a) DESeq false positive rates defined as the number of incorrectly identified DE genes

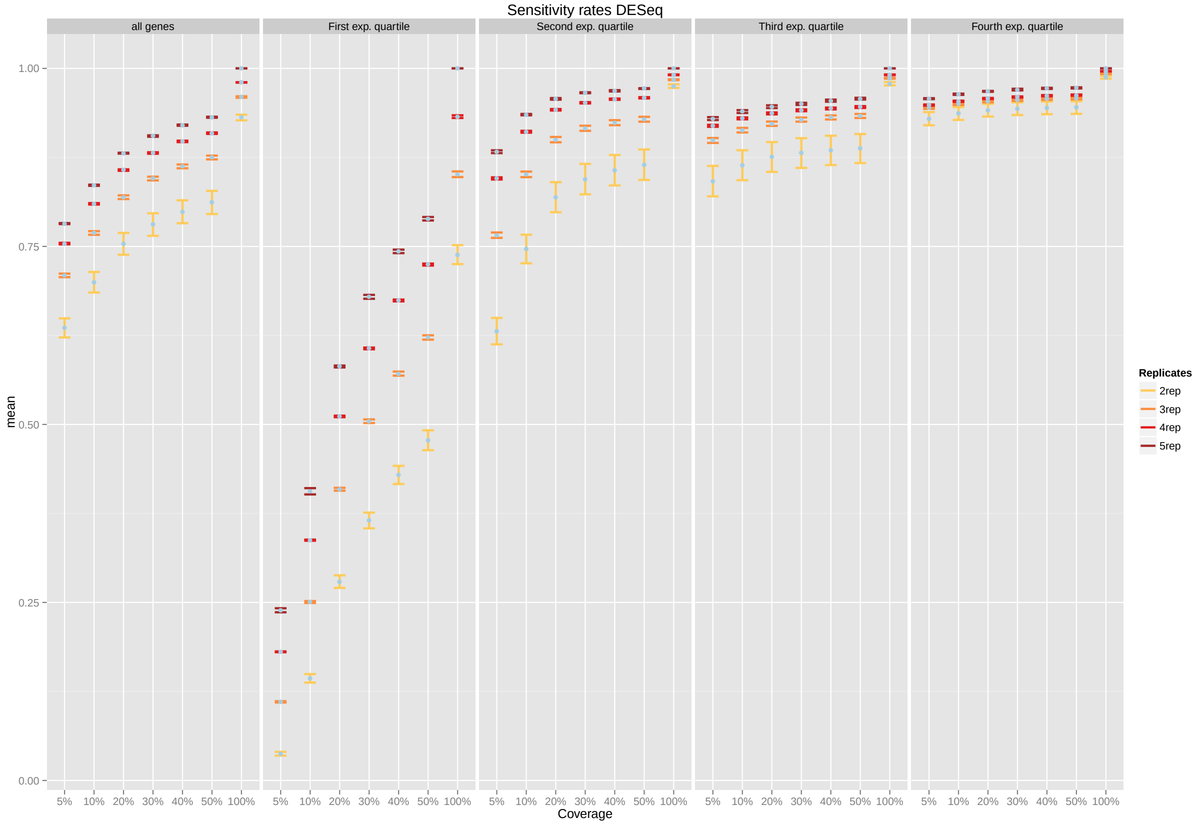

(b) DESeq sensitivity defined as the fraction of DE genes identified from the true set

Figure S 11: Impact of sequencing depth and number of replicate samples on DE detection by DESeq using SEQC data.

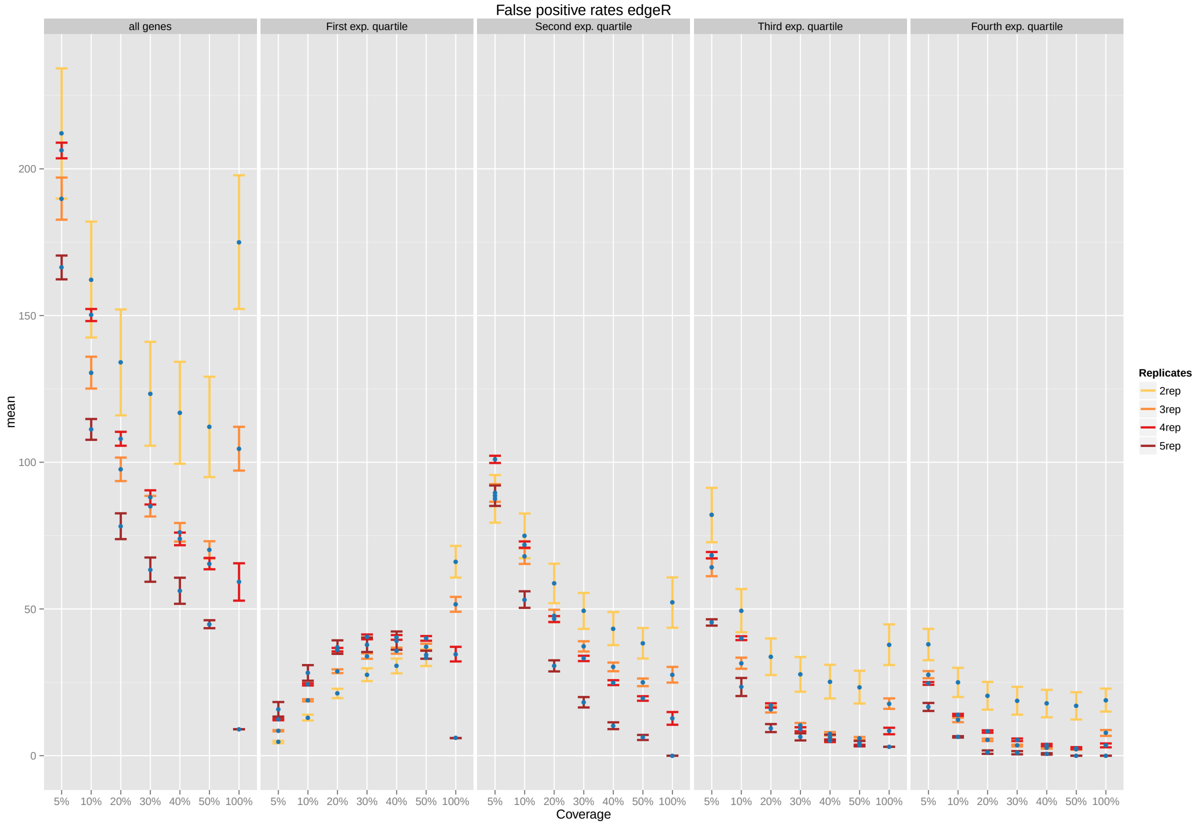

(a) edgeR false positive rates defined as the number of incorrectly identified DE genes

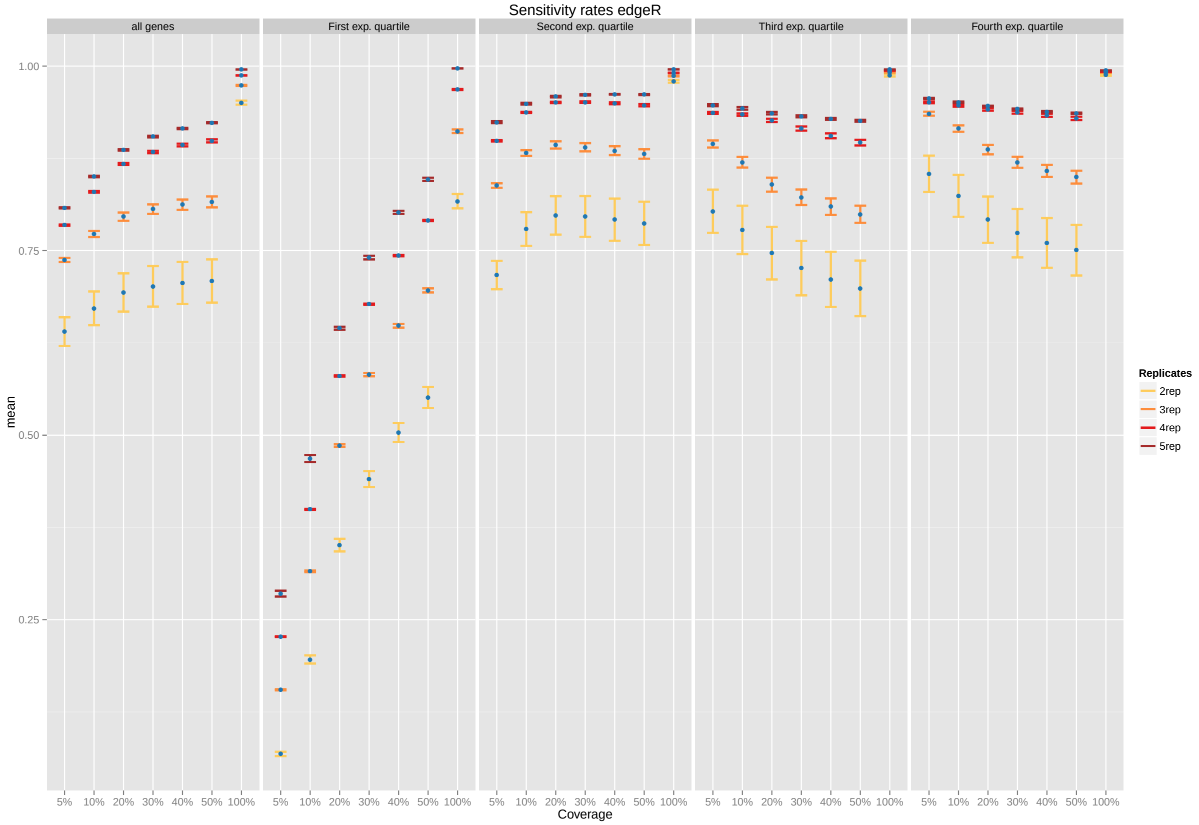

(b) edgeR sensitivity defined as the fraction of DE genes identified from the true set

Figure S 12: Impact of sequencing depth and number of replicate samples on DE detection by edgeR using SEQC data.

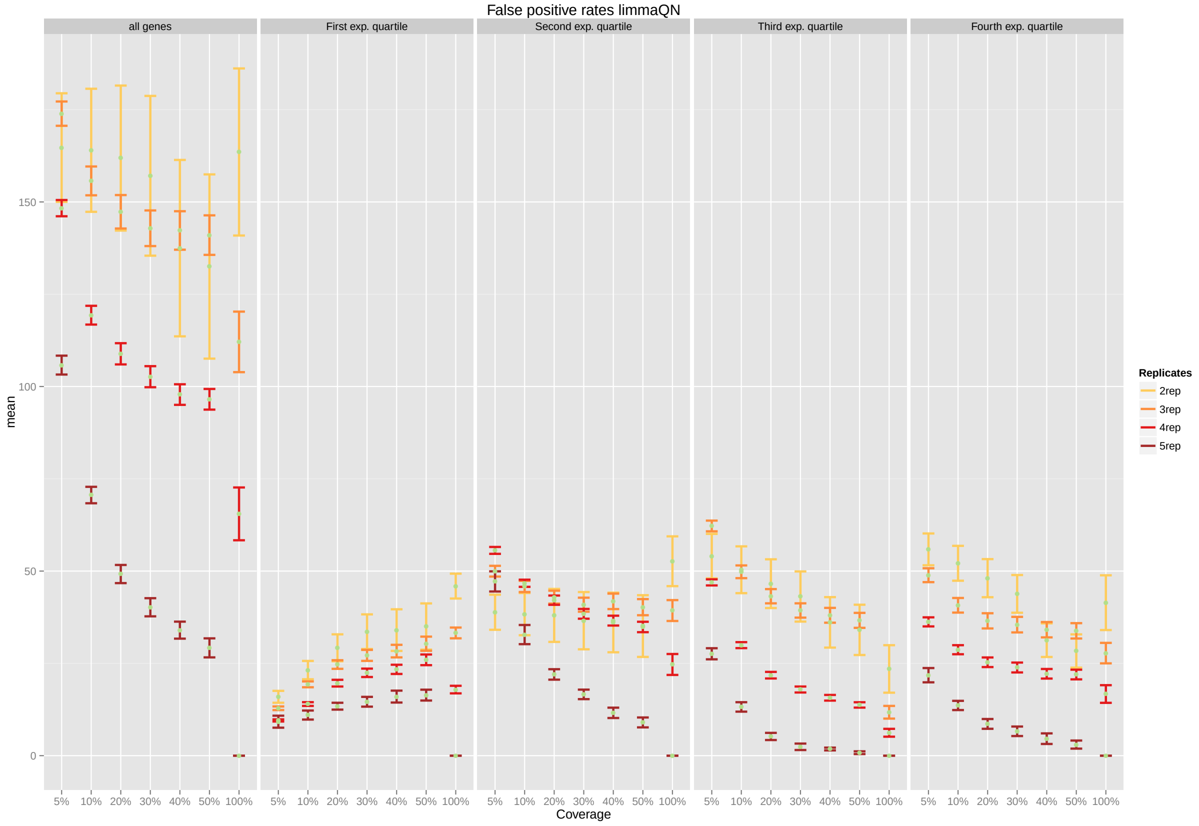

(a) limmaQN false positive rates defined as the number of incorrectly identified DE genes

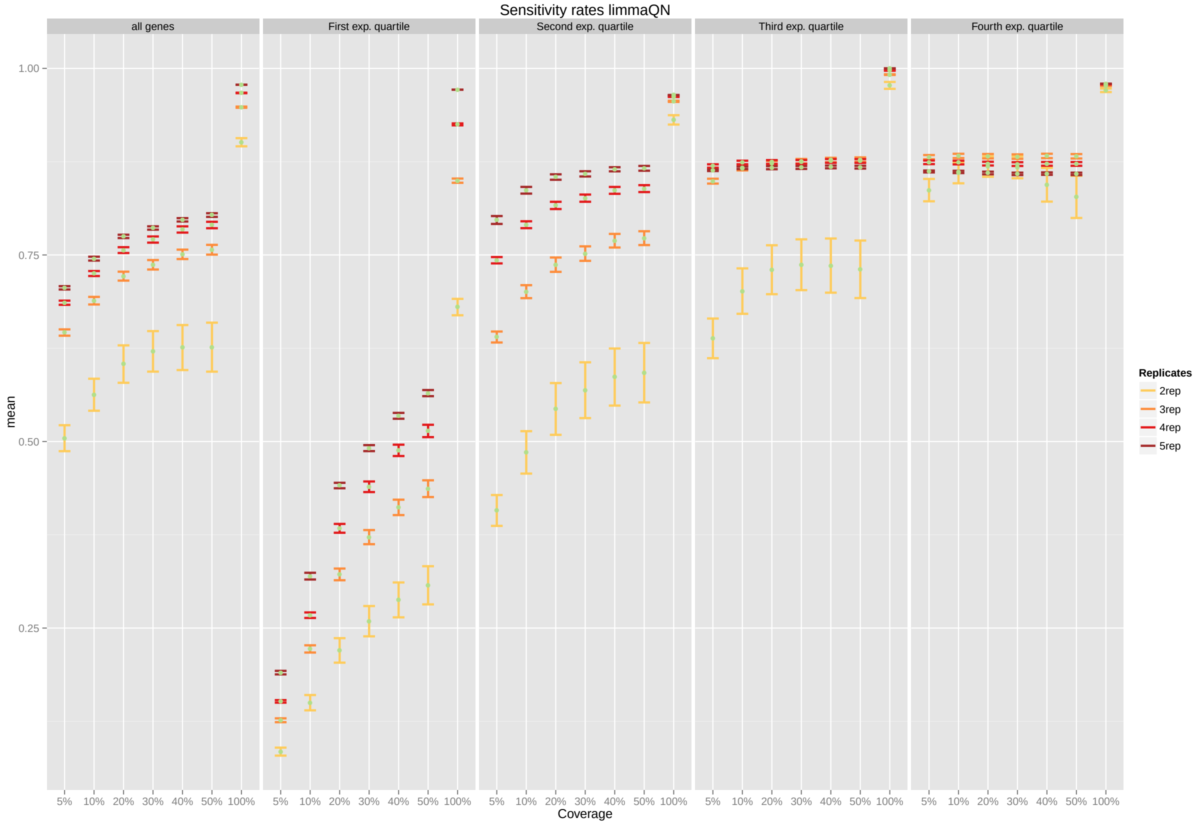

(b) limmaQN sensitivity defined as the fraction of DE genes identified from the true set. Note that limmaQN maximum sensitivity is less than 1 since limmaQN was not used to define the true set of DE genes.

Figure S 13: Impact of sequencing depth and number of replicate samples on DE detection by limmaQN using SEQC data.

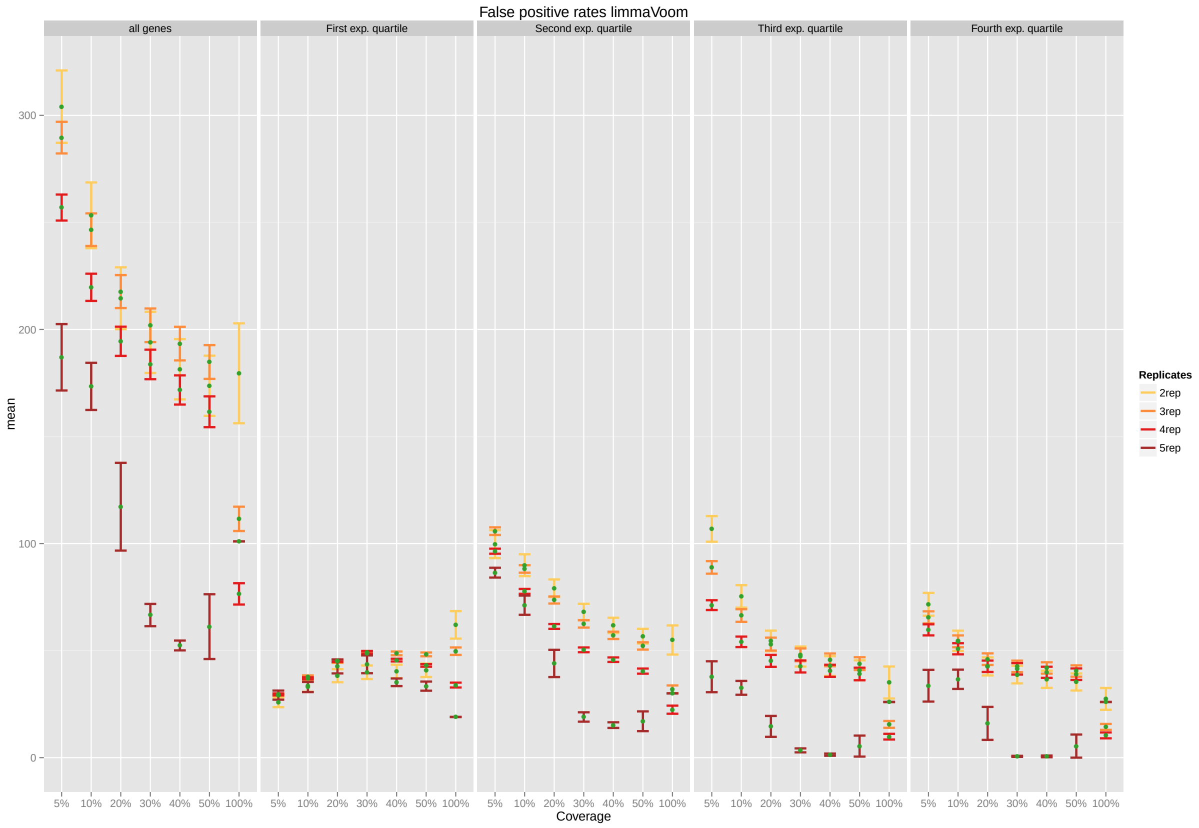

(a) limmaVoom false positive rates defined as the number of incorrectly identified DE genes

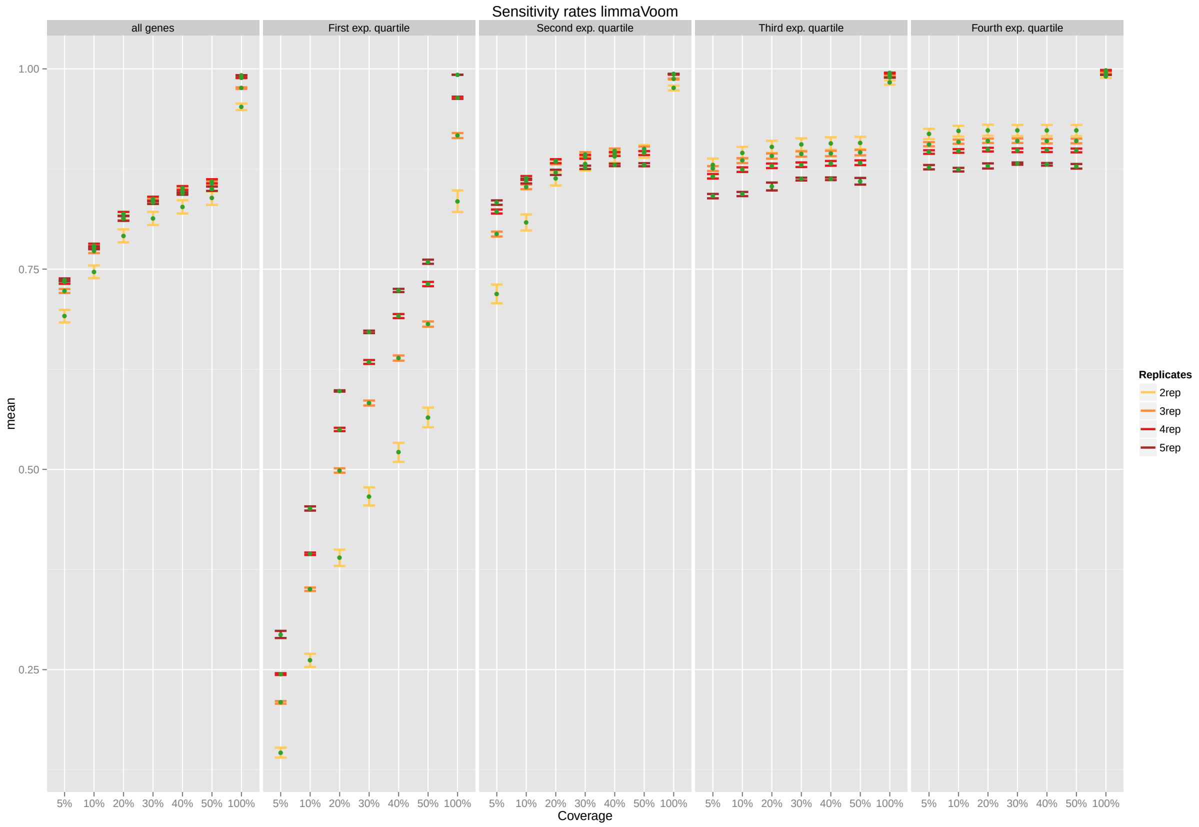

(b) limmaVoom sensitivity defined as the fraction of DE genes identified from the true set

Figure S 14: Impact of sequencing depth and number of replicate samples on DE detection by limmaVoom using SEQC data.

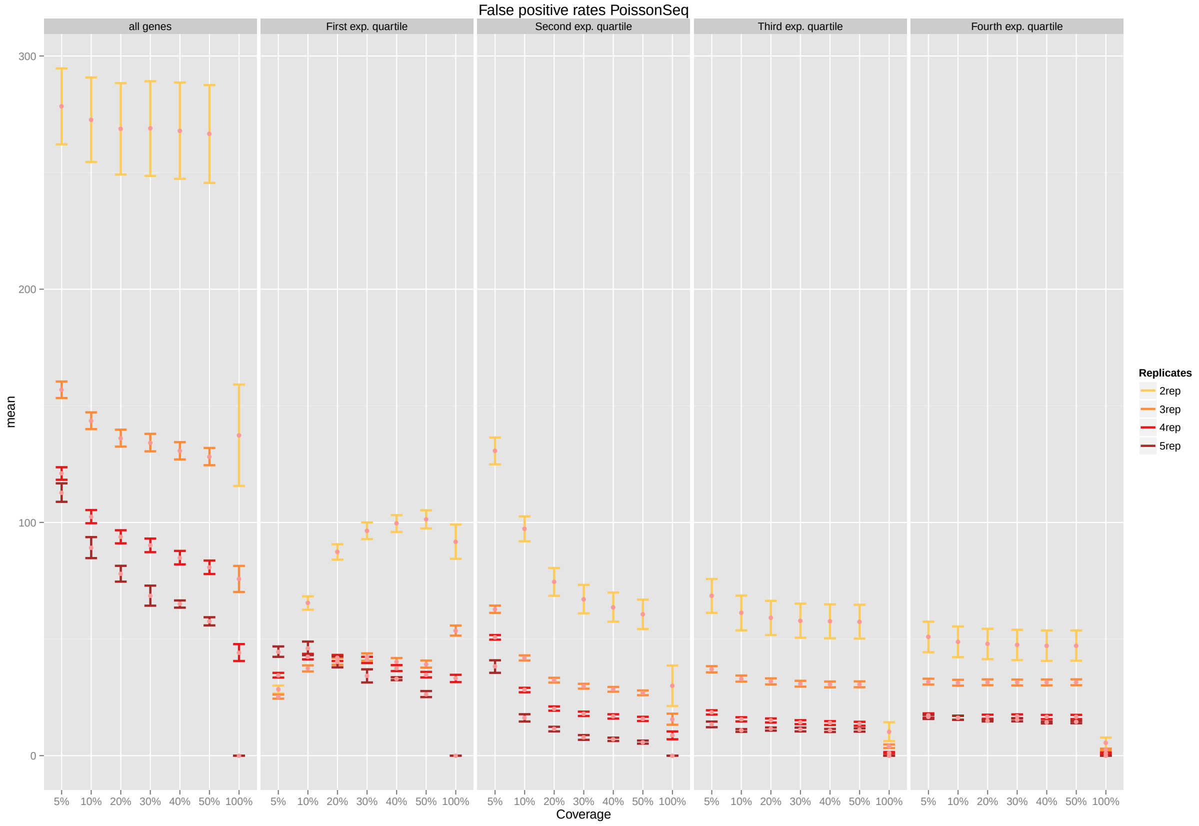

(a) PoissonSeq false positive rates defined as the number of incorrectly identified DE genes

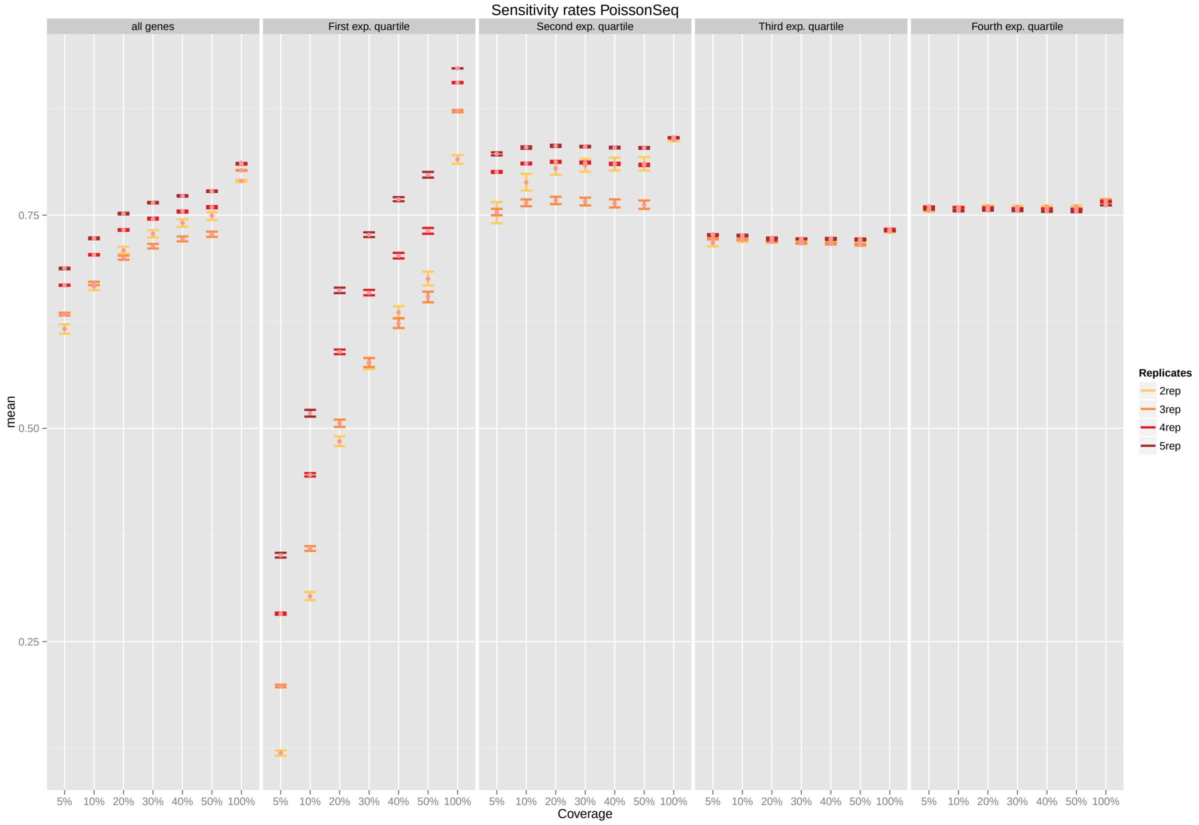

(b) PoissonSeq sensitivity defined as the fraction of DE genes identified from the true set. Note that PoissonSeq maximum sensitivity is less than 1 since PoissonSeq was not used to define the true set of DE genes.

Figure S 15: Impact of sequencing depth and number of replicate samples on DE detection by PoissonSeq using SEQC data.

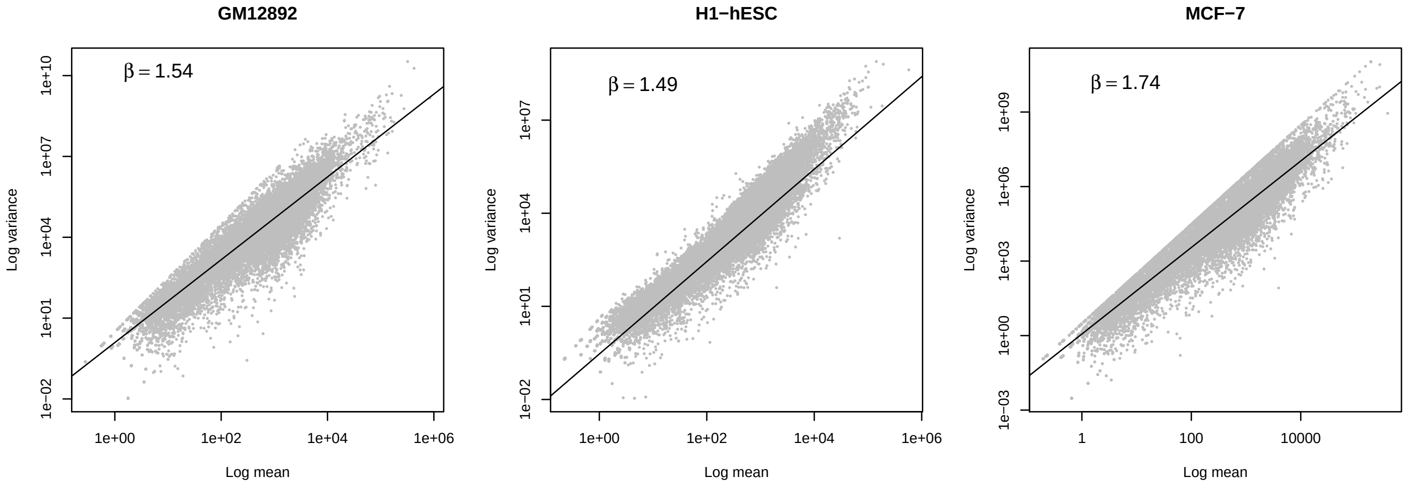

Figure S 16: **Over-dispersion of the ENCODE data set.** Over-dispersion indicates that the variance is larger than what would be expected given a specific model, for the case of count data, the Poisson model, in which the mean (μ) equals the variance (ν). In an over-dispersed data set, such as the ENCODE data, the mean-variance relation is super-linear $\nu \propto \mu^n$, where $n > 1$. In log space this would correspond to $\log \nu \propto n \log \mu$. The $\log \mu$ vs. $\log \nu$ plots for the three ENCODE cell lines used in this study are fitted with a linear regression line. The regression coefficients (β values in the plots) are all larger than one indicating that the variance is indeed increasing more rapidly than the mean and over dispersion exists among the ENCODE biological replicates.
